# Engineering canker‐resistant plants through CRISPR/Cas9‐targeted editing of the susceptibility gene *CsLOB1* promoter in citrus

**DOI:** 10.1111/pbi.12733

**Published:** 2017-05-03

**Authors:** Aihong Peng, Shanchun Chen, Tiangang Lei, Lanzhen Xu, Yongrui He, Liu Wu, Lixiao Yao, Xiuping Zou

**Affiliations:** ^1^ Citrus Research Institute Chinese Academy of Agricultural Sciences and National Center for Citrus Variety Improvement Chongqing China; ^2^ Citrus Research Institute Southwest University Chongqing China

**Keywords:** citrus canker, *CsLOB1*, genome editing, CRISPR/Cas9, resistance

## Abstract

Citrus canker, caused by *Xanthomonas citri* subsp. *citri* (*Xcc*), is severely damaging to the global citrus industry. Targeted editing of host disease‐susceptibility genes represents an interesting and potentially durable alternative in plant breeding for resistance. Here, we report improvement of citrus canker resistance through CRISPR/Cas9‐targeted modification of the susceptibility gene *CsLOB1* promoter in citrus. Wanjincheng orange (*Citrus sinensis* Osbeck) harbours at least three copies of the *CsLOB1* ^G^ allele and one copy of the *CsLOB1* ^−^ allele. The promoter of both alleles contains the effector binding element (EBE_P__thA4_), which is recognized by the main effector PthA4 of *Xcc* to activate *CsLOB1* expression to promote citrus canker development. Five pCas9/CsLOB1sgRNA constructs were designed to modify the EBE_P__thA4_ of the *CsLOB1* promoter in Wanjincheng orange. Among these constructs, mutation rates were 11.5%–64.7%. Homozygous mutants were generated directly from citrus explants. Sixteen lines that harboured EBE_P__thA4_ modifications were identified from 38 mutant plants. Four mutation lines (S2‐5, S2‐6, S2‐12 and S5‐13), in which promoter editing disrupted *CsLOB1* induction in response to *Xcc* infection, showed enhanced resistance to citrus canker compared with the wild type. No canker symptoms were observed in the S2‐6 and S5‐13 lines. Promoter editing of *CsLOB1* ^G^ alone was sufficient to enhance citrus canker resistance in Wanjincheng orange. Deletion of the entire EBE_P__thA4_ sequence from both *CsLOB1* alleles conferred a high degree of resistance to citrus canker. The results demonstrate that CRISPR/Cas9‐mediated promoter editing of *CsLOB1* is an efficient strategy for generation of canker‐resistant citrus cultivars.

## Introduction

Citrus canker, caused by *Xanthomonas citri* subsp. *citri* (*Xcc*), is one of the most destructive diseases causing severe yield losses in all citrus‐producing regions worldwide (Gottwald *et al*., 2002; Stover *et al*., 2014). Currently, the primary strategy for control of citrus canker relies on an integrated disease control approach, including production of disease‐free nursery stock, eradication programmes and use of antibiotics or bactericides (Gottwald *et al*., 2002; Graham *et al*., 2004). However, potential disadvantages of these methods include the high cost, risks to human and animal health, and adverse environmental effects. Breeding resistant cultivars is the most efficient and economical approach in the long term to control citrus canker. Various breeding strategies have been employed to produce disease‐resistant cultivars, among which genetic engineering remains the fastest method for improvement of existing citrus cultivars (Gong and Liu, 2013; Grosser *et al*., 2007).

In plant genetic breeding, resistance genes are usually used to improve plant resistance (van Schie and Takken, 2014). However, no active resistance genes have been identified in citrus because of the high degree of heterozygosity of citrus (Xu *et al*., 2013) and the wide host range of *Xcc* (Vojnov *et al*., 2010). This shortcoming hinders improvement of canker‐resistant citrus cultivars through a molecular breeding programme using resistance genes. To suppress or evade plant immunity, most pathogens require cooperation of the host to establish a compatible interaction. In this process, certain host genes are activated by the pathogen to favour pathogen growth and promote symptom development (Boch *et al*., 2014; Bogdanove *et al*., 2010; van Schie and Takken, 2014). All plant genes that facilitate infection and support compatibility are considered to be susceptibility genes (van Schie and Takken, 2014). Mutation of a susceptibility gene can render the host resistant to infection by the corresponding pathogen or even confer broad‐spectrum resistance (Blanvillain‐Baufume *et al*., 2017; Boch *et al*., 2014; Gawehns *et al*., 2013; Li *et al*., 2012; McGrann *et al*., 2014), which provides an interesting and alternative strategy in citrus breeding for resistance to citrus canker.

*LATERAL ORGAN BOUNDARIES 1* (*CsLOB1*), the susceptibility gene for citrus canker, plays a critical role in promoting pathogen growth and erumpent pustule formation (Hu *et al*., 2014). Recently, Jia *et al*. (2016b) reported that mutation of the coding region of *CsLOB1* in Duncan grapefruit (*Citrus × paradisi*) successfully generated citrus canker‐resistant plants. CsLOB1 belongs to the LBD (LOB Domain) family of proteins, which are key regulators of plant organ development (Xu *et al*., 2016). Therefore, the potential negative effect of mutating CsLOB1 function on plant development remains to be determined, although no phenotypic changes were observed in edited plants (Jia *et al*., 2016b). The main transcription activator‐like (TAL) effector of *Xcc*, PthA4, specifically binds to the effector binding element (EBE_PthA4_) in the *CsLOB1* promoter to activate expression of *CsLOB1* to favour citrus canker development (Hu *et al*., 2014; Li *et al*., 2014). *In vitro* tests show that mutation of the EBE_PthA4_ decreases and even abolishes the PthA4‐inducible activity of the *CsLOB1* promoter (Hu *et al*., 2014). As a result, *Xcc*‐inducible expression of *CsLOB1* is repressed. Moreover, for any susceptibility gene targeted by TAL effectors, an attractive possibility is to mutate the EBE in its promoter such that effector binding is abolished, but plant gene function remains intact (van Schie and Takken, 2014). In rice (*Oryza sativa* L.), genome editing of the EBEs of the susceptibility genes *Os11N3*,* Os14N3* and *Os12N3* confers resistance to bacterial blight via repression of pathogen‐induced gene expression (Blanvillain‐Baufume *et al*., 2017; Li *et al*., 2012; Zhou *et al*., 2015). Such mutations do not interfere with the developmental functions of the targeted genes (Li *et al*., 2012). Thus, editing of the EBE_PthA4_ of *CsLOB1* is a potential strategy for conferring resistance to citrus canker disease (Hu *et al*., 2014).

Recently, Jia *et al*. (2016a) reported on genome editing of the EBE_PthA4_ in the *CsLOB1* promoter for improvement of citrus canker resistance in Duncan grapefruit. However, all citrus mutants obtained in that study harboured only a 1‐bp insertion and no mutants displayed enhanced resistance to *Xcc*, which is the most widespread citrus canker pathogen in citrus‐growing regions worldwide (Gottwald *et al*., 2002). The mutations were insufficient to abolish TAL‐inducible expression of *CsLOB1* and thereby enhance plant disease resistance (Jia *et al*., 2016a). In contrast, modification of the EBEs of the rice susceptibility gene *OsSWEET14* confers resistance to bacterial blight via repression of gene expression, and in all resistant plants, more than four nucleotides were mutated in the EBEs (Blanvillain‐Baufume *et al*., 2017; Li *et al*., 2012). From the above‐mentioned results, we speculated that only PthA4 EBE mutations that repress or abolish the TAL‐inducible expression of *CsLOB1* may enhance plant disease resistance. Thus, as shown by Hu *et al*. (2014), mutation of additional nucleotides in the EBE_PthA4_ of *CsLOB1* may be required to confer citrus resistance to *Xcc*.

Targeted genome engineering, which allows the introduction of precise genetic modifications directly into a commercial cultivar, offers a powerful tool for plant genetic breeding. Among presently available genome‐editing technologies, the CRISPR/Cas9 system has been utilized widely for genome editing in many plant species, including citrus (Belhaj *et al*., 2015; Fan *et al*., 2015; Jia and Wang, 2014; Jia *et al*., 2016a,b; Ma *et al*., 2015; Samanta *et al*., 2016; Weeks *et al*., 2016). In this study, using CRISPR/Cas9 technology, we report improvement of citrus canker resistance via promoter‐targeted modification of the susceptibility gene *CsLOB1* in Wanjincheng orange (*Citrus sinensis* Osbeck). Of the mutant plants obtained, 42% showed modifications of the PthA4 EBE. Homozygous mutants were generated directly from infected citrus explants. Disease resistance and quantitative real‐time PCR (qPCR) analysis showed that genome editing of the *CsLOB1* promoter rendered modified plants resistance to citrus canker via disruption of *Xcc*‐mediated *CsLOB1* induction. This study provides an efficient approach for generation of canker‐resistant cultivars through modification of the *CsLOB1* promoter in citrus.

## Results

### Heterozygosity of the *CsLOB1* promoter in Wanjincheng orange

**Figure 1:**
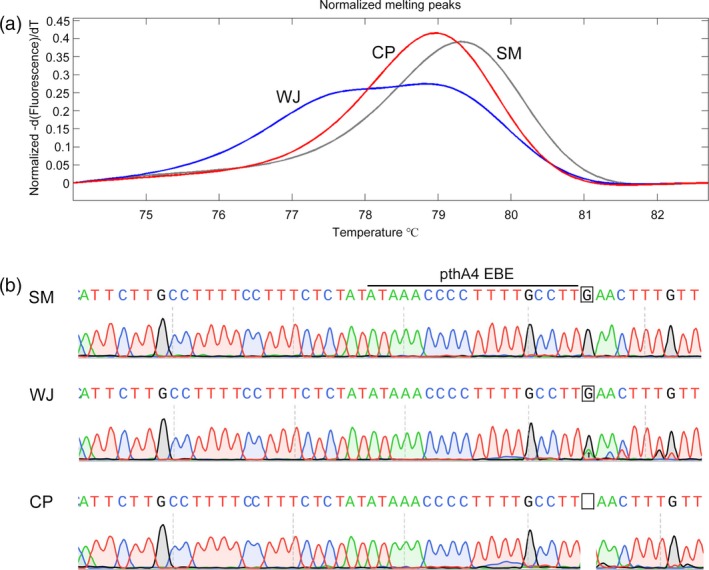
Genotype of the *CsLOB1* promoter in Wanjincheng orange (*Citrus sinensis* Osbeck). (a) High‐resolution melting analysis of the *CsLOB1* promoter in Wanjincheng orange (blue curve), using Chandler pummelo (*C. grandis*; red curve) and Satsuma mandarin (*C. unshiu*; grey curve) as controls. (b) Direct sequencing analysis of the *CsLOB1* promoter in Wanjincheng orange. Chromatograms for *CsLOB1* ^*G*^ */CsLOB1* ^*G*^, *CsLOB1* ^*G*^ */CsLOB1* ^*−*^ and *CsLOB1* ^*G*^ */CsLOB1* ^*−*^ in Satsuma mandarin (SM), Wanjincheng orange (WJ) and Chandler pummelo (CP) are shown. The indel tested is indicated by a square and arrow.

The *CsLOB1* promoter of sweet orange (*Citrus sinensis* Osbeck) ‘Valencia’ (Li *et al*., 2014; Xu *et al*., 2013) and Duncan grapefruit (Hu *et al*., 2014; Jia *et al*., 2016a) contains a G nucleotide at the first site after the 3′ end of the PthA4 EBE. In addition, sweet orange ‘Valencia’ (Abe and Benedetti, 2016; Hu *et al*., 2014) and Duncan grapefruit (Jia *et al*., 2016a) also harbour an allele of the promoter that lacks this nucleotide. In this study, on the basis of this indel (insertion/deletion), the *CsLOB1* allele containing the G nucleotide was designated *CsLOB1* ^*G*^ and the allele lacking this nucleotide was designated *CsLOB1* ^*−*^. To design efficient ‘single‐guide RNAs’ (sgRNAs) for CRISPR/Cas9‐induced modification of the *CsLOB1* promoter, the sequence characteristics of the promoter in the target material, Wanjincheng orange (*Citrus sinensis* Osbeck), were investigated in detail. Using Chandler pummelo (*Citrus grandis* Osbeck) and Satsuma mandarin [*Citrus unshiu* (Swingle) Marcow.] as controls, high‐resolution melting (HRM) analysis of the indel showed that three types of indel were present among the three species (Figure 1a). Direct sequencing analysis confirmed that Wanjincheng orange harboured both *CsLOB1* ^*G*^ and *CsLOB1* ^*−*^, whereas Satsuma mandarin and Chandler pummelo only carried *CsLOB1* ^*G*^ and *CsLOB1* ^*−*^, respectively (Figure 1b). The results indicated that *CsLOB1* ^*G*^ and *CsLOB1* ^*−*^ in Wanjincheng orange possibly originated from mandarin and pummelo, respectively (Xu *et al*., 2013). A ~500‐bp fragment of the *CsLOB* promoter, including the 5′ untranslated region, amplified from Wanjincheng orange was T‐cloned and subjected to sequencing. Sequencing analysis showed that the *CsLOB1* ^*G*^ and *CsLOB1* ^*−*^ promoters shared an identical PthA4 EBE sequence and, except for the G indel, the 40‐bp nucleotide sequence adjacent to the 5′ and 3′ ends of the EBE_PthA4_ was identical in the two types (Figure S1). Statistical analysis indicated that Wanjincheng orange contains at least three copies of *CsLOB1* ^*G*^ and one copy of *CsLOB1* ^*−*^ (Table S1), suggesting that this gene shows high heterozygosity in citrus. Other indels were also identified in the promoter (Figure S1).

### Efficient modification of the EBE induced by CRISPR/Cas9 in Wanjincheng orange

**Figure 2:**
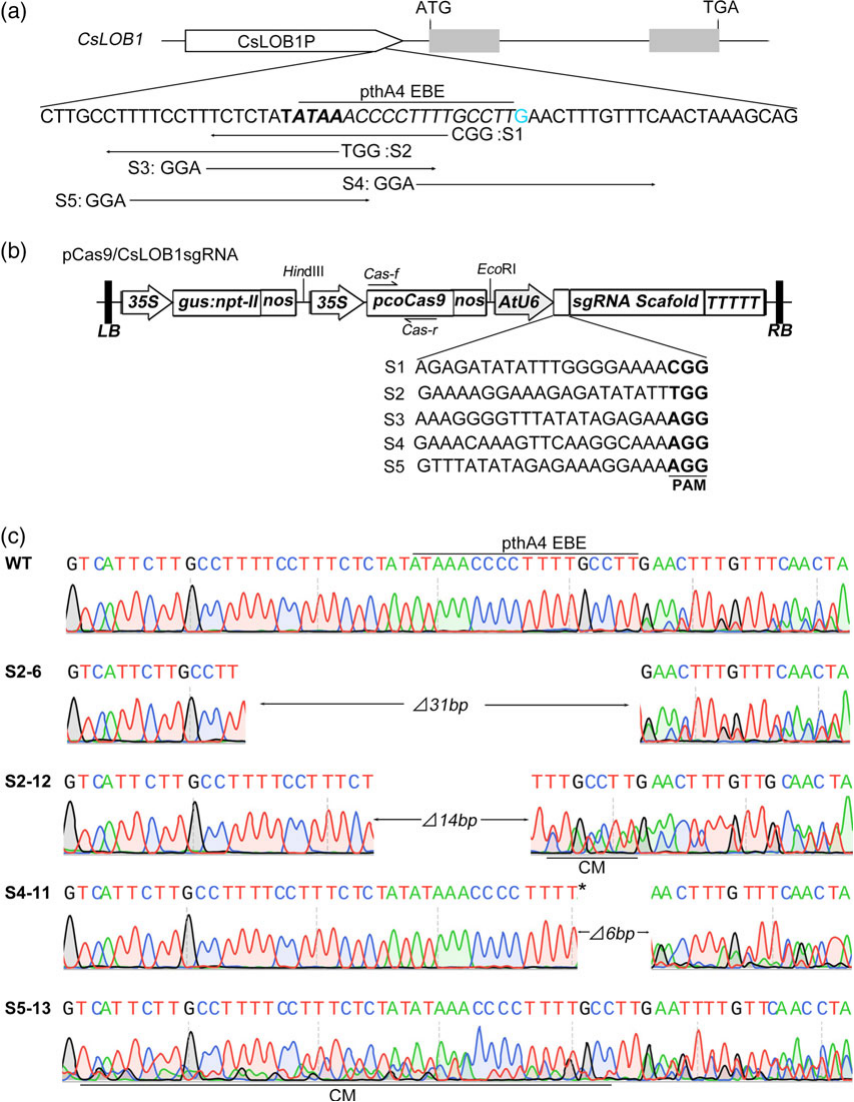
CRISPR/Cas9‐mediated modification of the *CsLOB1* promoter in Wanjincheng orange (*Citrus sinensis* Osbeck). (a) Schematic structure of *CsLOB1*. *CsLOB1* contains two exons indicated by grey rectangles. The translation initiation codon (ATG) and termination codon (TGA) are shown. The putative TATA box is in bold. The PthA4 effector binding element (EBE) sequence is in italics. The indel distinguishing *CsLOB1* ^*G*^ and *CsLOB1* ^*−*^ is in blue. The directions of sgRNAs (S1, S2, S3, S4 and S5) are indicated by long thin arrows. The protospacer adjacent motif (PAM) sites are shown. (b) Schematic diagram of pCas9/CsLOB1sgRNA vectors. 35S, *Cauliflower mosaic virus 35S* promoter from tobacco; *gus:npt‐II*, fusion of β‐glucuronidase and neomycin phosphotransferase genes; nos, nos terminator; *pcoCas9*, plant codon‐optimized *SpCas9* gene; AtU6, Arabidopsis U6‐1 polymerase III promoter; LB, left border; RB, right border. (c) Representative chromatograms of *CsLOB1* promoter mutations. ‘⊿#bp’ indicates the number of deleted nucleotides; ‘*’ indicates an insertion; ‘CM’ indicates chimera mutations.

Based on the sequence characteristics of the *CsLOB1* promoter in Wanjincheng orange, five sgRNAs were designed to target the PthA4 EBE (Figure 2a). Five corresponding Cas9/CsLOB1sgRNA plasmids were constructed (Figure 2b) and were transformed separately into the Wanjincheng orange genome by *Agrobacterium*‐mediated epicotyl transformation. In total, 110 independent transgenic plants were identified using β‐glucuronidase (GUS) histochemical staining and PCR analysis (Figure S2, Table S2). All of the transgenic plants were subjected to Sanger sequencing. Sequencing results showed that mutation rates were 11.5%–64.7% among the five sgRNA constructs, and a total of 38 transgenic plants showed mutations at the target sites (Table S2). These mutants comprised 28 chimera mutants, two biallelic mutants, two homozygous mutants and six heterozygous mutants (Tables 1, S3). Based on the allele mutation types, 60.5%, 20.9% and 18.6% of the mutations were deletions, insertions and substitutions, respectively (Table 2). All of the mutant plants carried modifications of *CsLOB1* ^*G*^, whereas only 31.6% of the mutant plants harboured modifications of *CsLOB1* ^*−*^ (Table S3). Sixteen of the targeted mutant plants harboured modifications in the EBE_PthA4_ region (Table S3). Among these 16 mutant plants, 1‐ to 50‐bp deletions were detected, and short (≤2 bp) insertions and substitutions were also observed (Figures 2c, S3).

**Table 1:** Proportions of mutant genotypes obtained with five sgRNAs in Wanjincheng orange (*Citrus sinensis* Osbeck)

sgRNA	Total no. of mutant genotypes	No. of chimeras^a^ (%)	No. of bialleles (%)	No. of homozygotes (%)	No. of heterozygotes (%)
S1	3	2 (66.7)	0 (0.0)	0 (0.0)	1 (33.3)
S2	10	6 (60.0)	2 (20.0)	2 (20.0)	0 (0.0)
S3	8	8 (100.0)	0 (0.0)	0 (0.0)	0 (0.0)
S4	11	6 (54.5)	0 (0.0)	0 (0.0)	5 (45.5)
S5	6	6 (100.0)	0 (0.0)	0 (0.0)	0 (0.0)
Total	38	28 (73.7)	2 (5.3)	2 (5.3)	6 (15.8)

For each sgRNA construct, the proportion of mutant genotypes (%) was calculated based on the number of each mutant genotype out of the total number of mutant genotypes. Ten clones per mutant line were sequenced to investigate the mutations.

a Chimera refers to a plant with at least three distinct alleles detected at the target site.

**Table 2:** Proportions of mutation types obtained with five sgRNAs in Wanjincheng orange (*Citrus sinensis* Osbeck)

sgRNA	Total no. of mutation types	No. of deletions (%)	No. of insertions (%)	No. of substitutions (%)
S1	4	1 (25.0)	1 (25.0)	2 (50.0)
S2	25	16 (64.0)	6 (24.0)	3 (12.0)
S3	22	12 (54.5)	7 (31.8)	3 (13.7)
S4	20	12 (60.0)	3 (15.0)	5 (25.0)
S5	14	10 (71.4)	1 (7.1)	3 (21.4)
Total	86	52 (60.5)	18 (20.9)	16 (18.6)

For each sgRNA construct, the proportion of mutation types (%) was calculated based on the number of each allele mutation type out of the total number of allele mutation types.

Large‐scale (more than 30 clones per mutant line) sequencing demonstrated that in individual mutant lines, the mutation rates of the EBE_PthA4_ in *CsLOB1* ^*G*^ and *CsLOB1* ^*−*^ were 8.8%–100% and 0%–100%, respectively (Table S4). The mutation rate of the EBE_PthA4_ was 100%, 86.0%, 32.5%, 90.7%, 83.8% and 32.4% for S2‐6, S2‐12, S4‐8, S4‐11, S4‐13 and S5‐13, respectively. Notably, the entire EBE_PthA4_ sequence was deleted from the genome of the homozygous mutant S2‐6 (Figure 2c). In the biallelic mutant S2‐5, a 182‐bp fragment just upstream of the EBE_PthA4_ was deleted from *CsLOB1* ^*G*^, although no modification in the EBE was detected (Figure S4). Thus, the line S2‐5 and the 16 lines containing modifications of the EBE_PthA4_ were selected for further analysis.

### Expression characteristics of *CsLOB1* in mutant plants

**Figure 3:**
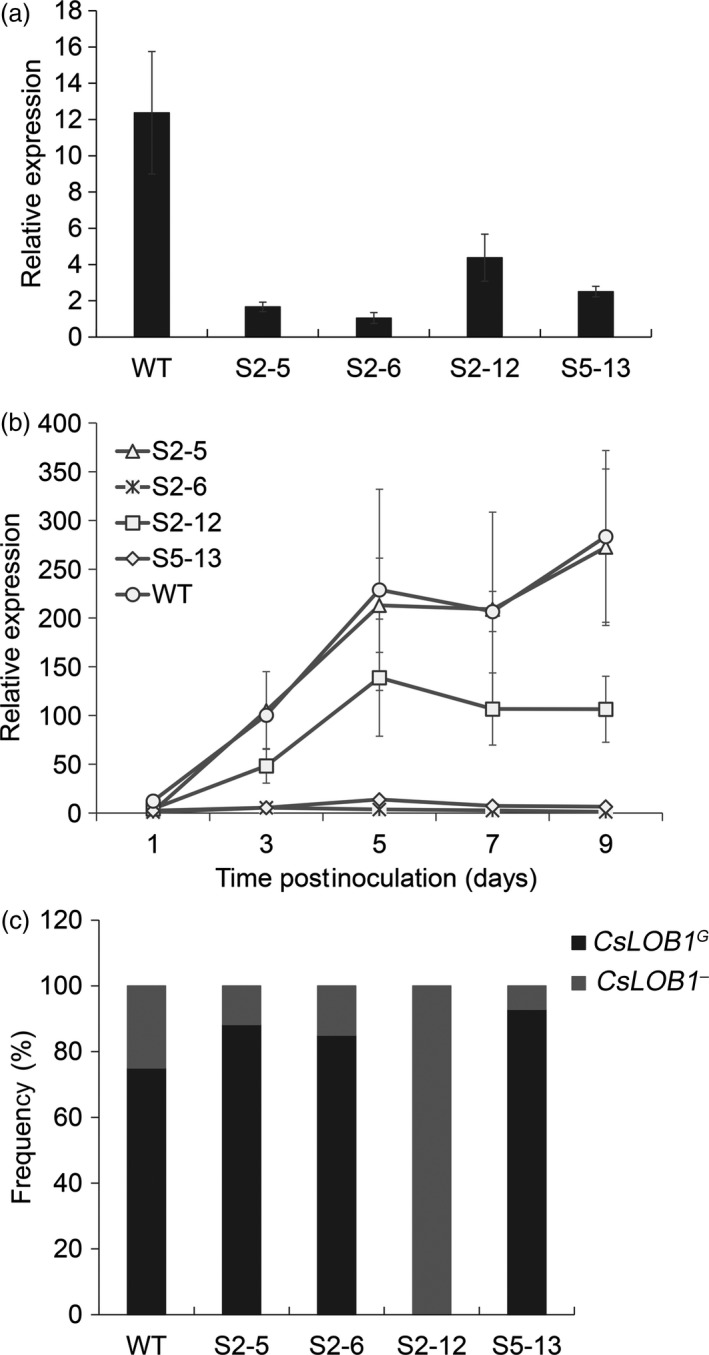
Expression characteristics of *CsLOB1* in Wanjincheng orange (*Citrus sinensis* Osbeck) mutants. (a) Expression of *CsLOB1* in mutant plants after *Xanthomonas citri* subsp. *citri* (*Xcc*) inoculation. At 1 day postinoculation (dpi), *CsLOB1* transcripts in leaves were analysed by quantitative real‐time PCR (qPCR). (b) Time course of *CsLOB1* expression in mutants after *Xcc* inoculation. Transcript levels of *CsLOB1* in leaves were determined by qPCR at 1, 3, 5, 7 and 9 dpi. (c) Statistical analysis of transcripts of *CsLOB1* ^*G*^ and *CsLOB1* ^*−*^ in citrus mutants. At 5 dpi, *CsLOB1* cDNA from infected leaves was amplified by PCR, cloned into the pGEM ^®^‐T Easy vector and sequenced. Twenty clones per mutant line were sequenced. Frequency (%) indicates the percentage of each *CsLOB1* mRNA out of the total mRNAs tested. Relative expression level of *CsLOB1* was determined by comparing the *CsLOB1* transcript levels after *Xcc* inoculation with that after water inoculation. Error bars (a and b) indicate standard deviation of three independent tests.

To determine the effects of EBE_PthA4_ modification on expression of *CsLOB1* in the mutant plants, we investigated the *CsLOB1* expression characteristics in mutant lines after inoculation with *Xcc*. At 1 day postinoculation (dpi), the relative expression level of *CsLOB1* in most mutant lines showed no obvious difference from that of the wild type (Figure S5). However, in S2‐5, S2‐12 and S5‐13, the relative expression level of *CsLOB1* was markedly lower than that of the wild type and no inducible expression was detected in the line S2‐6 (Figure 3a). At 3–9 dpi, the relative expression level of *CsLOB1* in S2‐5 and S2‐12 remained lower than that of the wild type, although no significant difference was detected compared with the wild type (Figure 3b). For S2‐6 and S5‐13, the pathogen‐inducible expression levels of *CsLOB1* were considerably lower than that of the wild type during the inoculation period (Figure 3b). These results showed that the mutations harboured in these lines successfully repressed expression of *CsLOB1* activated by *Xcc* infection.

**Figure 4:**
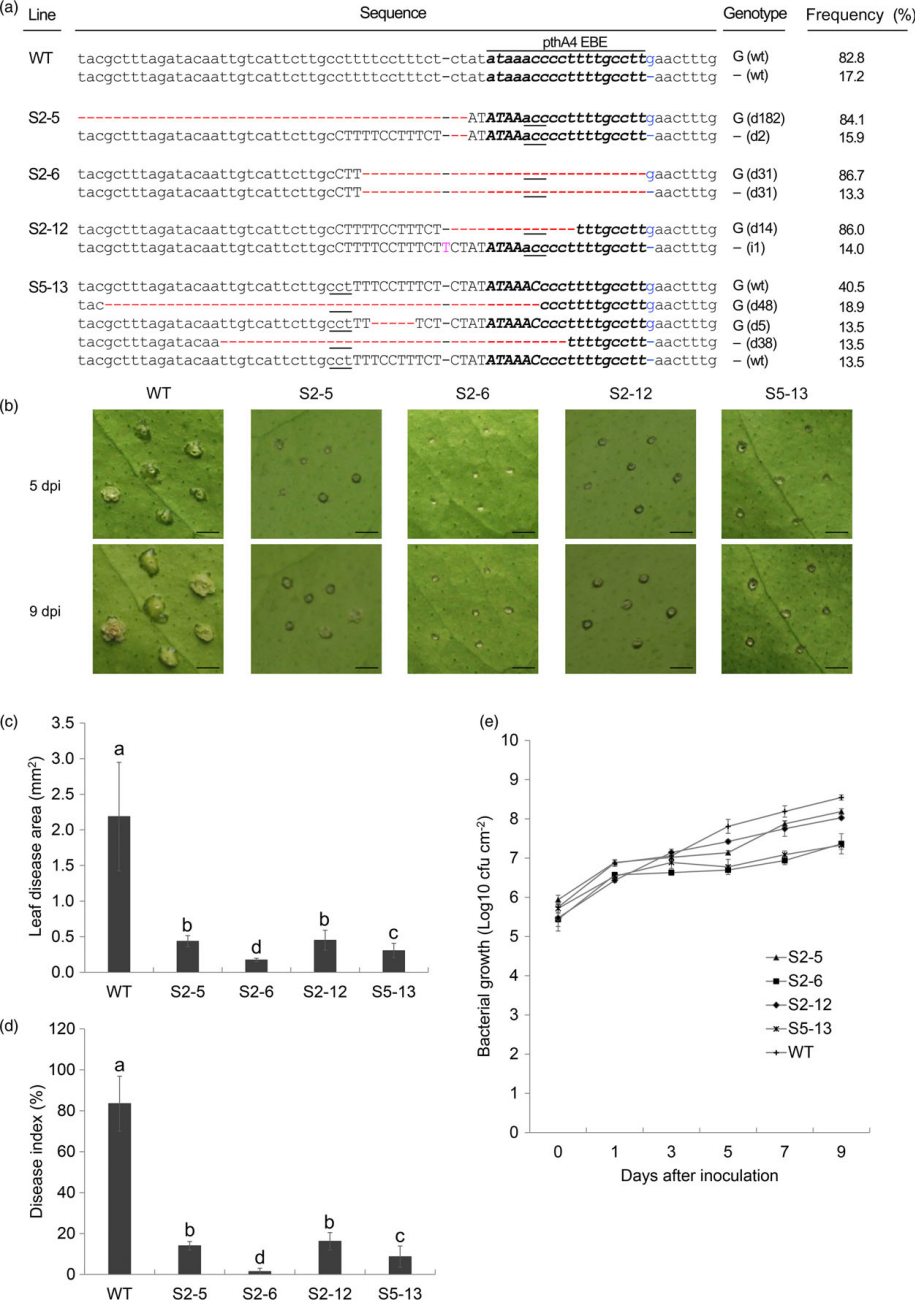
Identification of citrus canker resistance in Wanjincheng orange (*Citrus sinensis* Osbeck) mutants. (a) Representative sequences of *CsLOB1* mutations induced by CRISPR/Cas9. The pthA4 effector binding element (EBE) is in bold italics. The sgRNAs are in upper‐case letters, and the protospacer adjacent motif (PAM) site is underlined. The indel distinguishing *CsLOB1* ^*G*^ and *CsLOB1* ^*−*^ is in blue. Red dashes indicate deleted nucleotides. Pink letters indicate inserted nucleotides. ‘G ()’and ‘– ()’indicate mutations of *CsLOB1* ^*G*^ and *CsLOB1* ^*−*^, respectively. In parentheses, ‘d#’ and ‘i#’ indicate the number of nucleotides deleted and inserted, respectively. Frequency (%) was calculated based on the number of clones with the same mutation out of the total number of clones sequenced. More than forty clones per line were sequenced to investigate mutations. (b, c and d) Assay of resistance to *Xanthomonas citri* subsp*. citri* (*Xcc*) in mutant plants. Fully expanded leaves of mutant lines and the wild type were treated with 10^5^ CFU/mL *Xcc*. Citrus canker symptoms (b) were recorded by photographing 5 and 9 days postinoculation (dpi). Disease lesion area (c) and disease index (d) of leaves of each mutation line were investigated at 9 dpi. (e) Growth of *Xcc* in leaves of mutant plants. Values are expressed as means ± standard deviation of six independent experiments. Different letters above bars represent significant differences from the wild type based on Duncan's multiple range test (*P *<* *0.05). WT, wild type. In (b), bars = 1 mm.

The 714‐bp transcripts of *CsLOB1* ^*G*^ and *CsLOB1* ^*−*^ containing the coding sequence (Figure S6) were further investigated in the mutants by sequencing analysis (Figure 3c). In S2‐5, S2‐6 and S5‐13, the proportions of mRNAs of *CsLOB1* ^*G*^ and *CsLOB1* ^*−*^ were similar to those of the wild type, in which most transcripts detected were *CsLOB1* ^*G*^ mRNAs. No transcripts of *CsLOB1* ^*G*^ were detected in the biallelic mutant S2‐12, which was consistent with the occurrence of EBE_PthA4_ mutation only in *CsLOB1* ^*G*^ (Figure 4a). These results showed that *CsLOB1* ^*G*^ response to *Xcc* infection is dominant in Wanjincheng orange.

### Mutant plants show enhanced resistance to citrus canker

To characterize the canker resistance of the citrus mutants, the line S2‐5 and the 16 lines containing modifications of the EBE_PthA4_ were first evaluated for resistance to *Xcc* by pinprick inoculation. Disease development was determined at 5 and 9 dpi (Figures 4b, S7). In S2‐5 and S2‐12, eruption of pustules on the leaf surface was much slower than that of the wild type and, consequently, diseased lesions were markedly smaller than those in the wild type. Narrow rings of pustules around the puncture sites were observed in S5‐13 and no pustules were detected in S2‐6 at 5 dpi. At 9 dpi, some cells around the puncture sites showed hypertrophy in S2‐6. During the inoculation period, no pustules in S2‐6 and S5‐13 developed into typical cankers, whereas a few small cankers were observed in S2‐5 and S2‐12 (Figures 4b, S7). For the wild type, typical canker symptoms developed at all inoculation sites. These observations showed that mutations in the *CsLOB1* promoter inhibited the development of pustules induced by *Xcc* infection, and line S2‐6 showed the strongest resistance to canker development.

Statistical analysis showed that diseased lesions in S2‐5, S2‐6, S2‐12 and S5‐13 were significantly smaller than those in the wild type at 9 dpi (Figure 4c). The lesion area in S2‐6 and S5‐13 was 0.17 ± 0.02 mm^2^ and 0.30 ± 0.10 mm^2^, respectively, which was not significantly different from the puncture size of the inoculating pin (0.20 mm^2^); thus, pathogen spread on the leaf surface was entirely suppressed. Other mutants showed no difference in area of the diseased lesion compared with the wild type (Figure S8). Estimation of disease severity (Figure 4d) revealed that the disease index of S2‐5 (14.0%), S2‐6 (1.5%), S2‐12 (16.2%) and S5‐13 (8.7%) was significantly lower than that of the wild type (83.5%) at 9 dpi. The disease severity of these lines was reduced by 83.2%–98.3% compared with that of the wild type. Bacterial growth assay showed that *Xcc* populations in the four mutant lines were similar to that in the wild type up to 1–4 dpi (Figure 4e). After that time, *Xcc* populations in mutant plants were significantly smaller than that in the wild type (Figure 4e), indicating that bacterial growth in mutant plants was inhibited.

**Figure 5:**
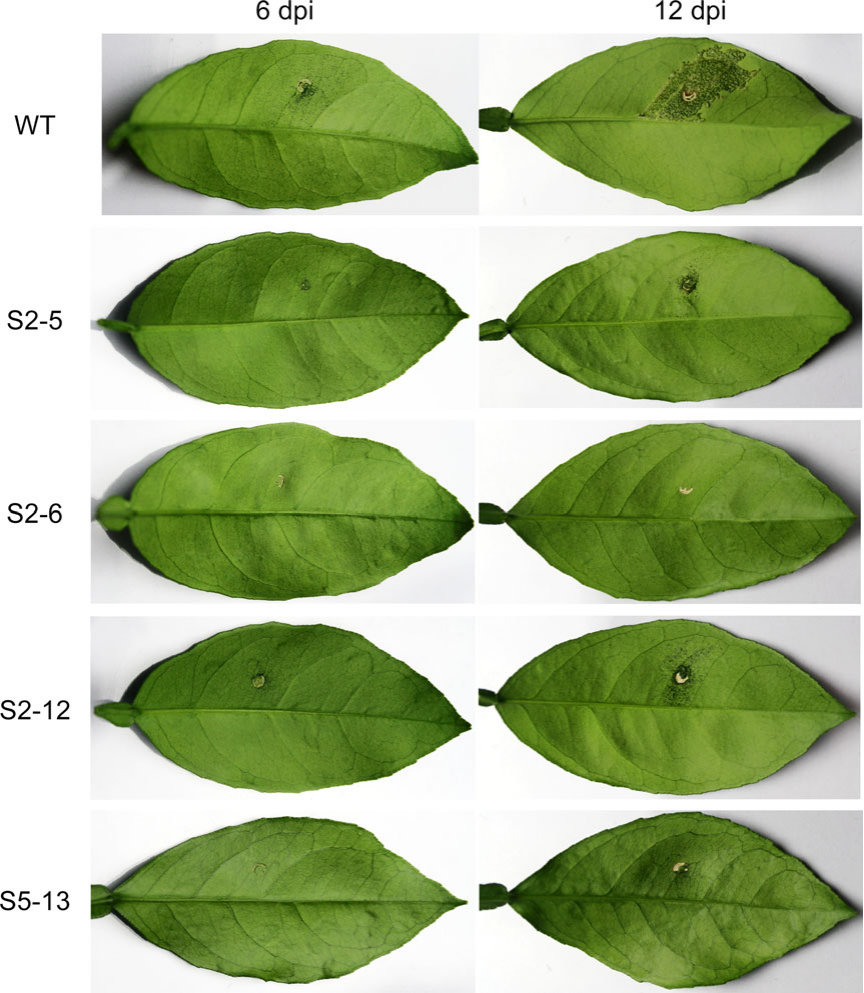
*In vivo* assay of citrus canker resistance in Wanjincheng orange (*Citrus sinensis* Osbeck) mutants. Leaves were infiltrated with *Xanthomonas citri* subsp. *citri* (*Xcc*) suspensions. At 6 days postinoculation (dpi), pustules were detected in wild type, but absent or significantly reduced in mutant plants. At 12 dpi, severe canker symptoms were detected in wild type, whereas markedly reduced symptoms were observed in S2‐5 and S2‐12. No canker symptoms were found in S2‐6 and S5‐13.

Resistance of edited plants to canker citrus was further confirmed using *in vivo* infiltration (Figure 5). No pustules or canker symptoms were detected in S2‐6 up to 12 dpi. In S2‐5 and S2‐12, pustules and canker symptoms were significantly reduced compared to that in the wild type during the inoculation. A few small pustules were found in S5‐13 at 12 dpi. A comparison of these results with those of the *in vitro* assay (Figure 4) revealed that these four edited lines had strong and stable resistance to citrus canker and the line S2‐6 showed the highest degree of resistance.

### Off‐target mutation analysis

The potential off‐target effect of CRISPR/Cas9 was evaluated in S2‐5, S2‐6, S2‐12 and S5‐13 by Sanger sequencing. Employing the CRISPR‐P Web tool (Lei *et al*., 2014), 205 putative off‐targets mediated by the S2 and S5 sgRNAs were detected in the citrus genome. Eleven putative off‐target loci, which contained a protospacer adjacent motif (PAM) (NGG or NAG) sequence and showed high sequence similarity to the S2 and S5 target sites, were chosen for further examination of potential off‐target effects (Tables 3, S5). Mutations in all of the putative off‐target loci were detected in the mutant lines tested. However, the off‐target frequencies were low (5.0–10.0%) and all of the mutations consisted of 1‐bp point mutations.

**Table 3:** Mutation analysis of putative CRISPR/Cas9 off‐target sites in Wanjincheng orange (*Citrus sinensis* Osbeck)

Line	Name of putative off‐target site (No. of clones with mutation/Total no. of clones tested)										
	O1	O2	O3	O4	O5	O6	O7	O8	O9	O10	O11
S2‐5	0/16	0/17	0/20	2/20	0/20	2/21	1/16	0/19	0/20	0/22	0/20
S2‐6	0/20	0/20	0/22	0/20	0/21	2/22	2/20	0/18	0/19	0/16	0/17
S2‐12	0/20	0/15	0/19	1/20	1/17	1/20	1/21	0/16	0/16	0/19	0/22
S5‐13	2/23	0/18	0/18	0/17	0/16	2/20	1/16	2/21	0/17	0/20	0/19

## Discussion

The global citrus industry faces many biotic and abiotic challenges, including bacterial canker and citrus greening disease. Screening for targeted mutants is a useful strategy for plant functional genomics research and for crop improvement. In citrus, however, this approach is problematic for research on gene function and genetic improvement because of phenomena such as male/female sterility, the long juvenile phase, the high degree of heterozygosity and polyembryony (Gong and Liu, 2013; Grosser *et al*., 2007). Targeted genome‐editing technologies, which can precisely and efficiently induce specific mutations, has been used in plant molecular research and for genetic improvement of crops (Li *et al*., 2015). Previous studies have proven that the CRISPR/Cas9 system induces mutations in citrus (Jia and Wang, 2014; Jia *et al*., 2016a,b). Using *CsLOB1* as the target gene, we accumulated comparative data on CRISPR/Cas9‐induced mutation efficiencies, mutation types and cleavage specificity in citrus. A high efficiency of recovery (maximum 64.7%) of mutant plants was achieved, which is similar to that reported for other major crops (Osakabe and Osakabe, 2015). We observed that 42.0% of the mutant plants harboured the desired modifications and 23.5% of these mutants showed resistance to citrus canker. The present study demonstrated that the CRISPR/Cas9 system directly generated homozygous targeted mutations in the first regenerated shoots, which is extremely important for genetic modification of asexually propagated citrus, which is characterized by a long juvenile phase and high heterozygosity. With the advantages of both high editing efficiency and homologous mutation, citrus mutants in genes of interest could be generated rapidly (in approximately one month). In addition, mutation frequencies of potential off‐target sites were extremely low. Taken together, the present results show that CRISPR/Cas9‐induced mutagenesis is precise and efficient in citrus, which will help to accelerate basic research and genetic improvement in citrus.

CRISPR/Cas9‐mediated genome editing usually generates five potential genotypes, namely homozygous, biallelic, heterozygous and chimera mutants and the wild type (Pan *et al*., 2016; Zhang *et al*., 2014). All of the five genotypes were detected in the present study. Compared with other crops (Feng *et al*., 2014; Ma *et al*., 2015; Wang *et al*., 2016; Xu *et al*., 2015), a high percentage (73.7%) of chimera mutants was observed, whereas only 5.3% of mutants were biallelic or homozygous in our study (Table 1). Similarly, a high percentage of chimera mutants was detected in Duncan grapefruit (Jia *et al*., 2016a,b). Chimeric mutation is considered to arise after division of the transformed cell (Zhang *et al*., 2014). In citrus stem transformation, most of the cells competent for transformation are the actively dividing cells located in the cambial ring of explants (Peña *et al*., 2004). Transformed cells undergoing division do not offer sufficient time for the sgRNA/Cas9 complex to edit all copies of a targeted gene before the first division. In contrast, the continuous activity of the Cas9/sgRNA complex during shoot generation may give rise to the high frequency of chimeric mutations. In addition, chimeric transgenic shoots are frequently generated from fusion of transformed and nontransformed cells, and even from fusion of different transformed cells in citrus stem transformation (Domínguez *et al*., 2004). As a result, in different genome loci, in which the CRISPR/Cas9 system was integrated, the expression of Cas9 and sgRNA may differ among transformed cells in a single transgenic plant. The expression levels of Cas9 and sgRNA are important factors that affect editing efficiency (Wu *et al*., 2014). Thus, this transgenic chimera may favour CRISPR/Cas9‐mediated chimeric mutation. Finally, most of citrus varieties are heterozygous diploids showing high heterozygosity. We speculate that this type of heterozygosity may affect the efficacy of the CRISPR/Cas9 system, probably resulting in the high frequency of chimera mutants. For example, most mutations were induced at the *CsLOB1* ^*G*^ locus, indicating that the *CsLOB1* ^*G*^ locus is more susceptible to Cas9/sgRNA editing. In future studies, increased effort to prohibit CRISPR/Cas9‐mediated chimeric mutation in citrus is required.

It has been suggested that mutation in both alleles of *CsLOB1* is required to generate citrus canker‐resistant plants (Jia *et al*., 2016a,b). Similarly, our data showed that deletion of the entire EBE_PthA4_ sequence from both *CsLOB1* alleles conferred the highest level of resistance to citrus canker (Figure 4). Five (A, A*, Aw, B and C) representative *Xanthomonas* strains contain distinct TAL effectors, pthA4, pthA*, pthAw, pthB and pthC, respectively, which are essential for pustule formation on citrus (Al‐Saadi *et al*., 2007). All the effectors can induce the expression of *CsLOB1* in sweet orange and grapefruit (Hu *et al*., 2014). The EBEs of pthA* and pthAw were located at the same position as that of PthA4, while the EBEs of PthB and PthC overlaps and starts 6 bp upstream of the EBE_PthA4_ (Hu *et al*., 2014). Abe and Benedetti (2016) show that PthA1, PthA2 and PthA3 from *Xcc* appear to have overlapping EBEs in promoters of canker susceptibility genes. Thus, it is predictable that deletion of the entire EBE_PthA4_ sequence or larger deletion comprising the EBE region from both *CsLOB1* alleles will render edited plants broad‐spectrum resistance to most kinds of citrus canker (Hu *et al*., 2014).

Moreover, some differences in the possible resistance mechanisms of *CsLOB1* modifications were observed in edited plants. (1) S2‐12 and S2‐5 showed that mutation of *CsLOB1* ^*G*^ alone is sufficient to enhance citrus canker resistance, which indicated that *CsLOB1* ^*G*^ is a dominant allele in TAL‐induced *Xcc* virulence in Wanjincheng orange. This conclusion is supported by our DNA and mRNA sequencing analyses (Table S1, Figure 3c). Jia *et al*. (2016a) reported that the ratio of *CsLOB1* ^*G*^ to *CsLOB1* ^*−*^ is 1:1 in Duncan grapefruit, whereas in the current study, the ratio was 3:1 in Wanjincheng orange (Table S1). Satsuma mandarin and Chandler pummelo only harbour one type of *CsLOB1* (Figure 1). This polymorphism is also detected in other citrus varieties (Abe and Benedetti, 2016). These data indicate that *CsLOB1* genes are complex in citrus, which may influence the roles of *CsLOB1* ^*G*^ and *CsLOB1* ^*−*^ in different cultivars. In addition, high degrees of heterozygosity and genetic differences are evident among citrus cultivars (Gmitter *et al*., 2012; Xu *et al*., 2013). Thus, editing of *CsLOB1* genes for citrus canker resistance should be explored on a case‐by‐case basis in citrus. (2) Surprisingly, the S5‐13 chimera mutant showed a high level of resistance and no citrus canker symptoms, although only 32.4% of the modified EBE_PthA4_ was present in the *CsLOB1* promoter (Figure 4). We speculate that this mutation occurred possibly in a specific cell layer, such as the L1 cell layer. The plant epidermis originates from the L1 cell layer and is an important early barrier to pathogen infection. *Xcc* cells within citrus tissues must rupture the epidermis to form a canker (Brunings and Gabriel, 2003), and pthA4 mutants of *Xcc* do not induce pustule formation on the epidermis (Hu *et al*., 2014). These data indicate that the epidermis plays a crucial role in inhibiting canker development. Therefore, *CsLOB1* mutation in the epidermis cells might be sufficient to confer high resistance. (3) In S2‐5, the deletion of the 182‐bp sequence upstream of the EBE_PthA4_ from *CsLOB1* ^*G*^ enhanced plant resistance to citrus canker. TAL effector‐mediated activation of host target genes requires certain accessible host helper proteins, for example, the general transcription factor TFIIA, in which mutation can inhibit exploitation by TAL effectors to activate host target genes (Gu *et al*., 2009). The 182‐bp sequence is predicted to contain specific *cis*‐elements (such as the CAAT box; Figure S1) for binding of similar transcription factors or other TAL effectors that are required for *Xcc* to activate *CslOB1* expression.

The mutation rates (32.5%–90.7%) of the PthA4 EBE in S4‐8, S4‐11 and S4‐13 were comparable to that (32.4%–100%) of the canker‐resistant lines S2‐6, S2‐12 and S5‐13 (Figures 4a, S3). However, these lines have no enhanced resistance to citrus canker (Figure S8). Many EBEs overlap with or encompass the TATA box, which is critical for transcriptional regulation (Hu *et al*., 2014; Pereira *et al*., 2014), and modification of the TATA box can decrease or even eliminate gene expression (Antony *et al*., 2010; Hu *et al*., 2014). Further analysis revealed that S2‐6, S2‐12 and S5‐13 had deletion of the TATA box (Figure 4a), whereas S4‐8, S4‐11 and S4‐13 lacked this mutation (Figure S3). These data indicate that the TATA box plays an important role in the interaction of TAL effectors and target genes and that mutants with TATA box deletion should be one main objective for citrus canker resistance breeding. S2‐8, S2‐9, S3‐5 and S5‐3 showed no resistance (Figure S8) although deletion of the TATA box was detected (Figure S3). This was due to low deletion rates (7.5%–21.2%) of the TATA box in these lines (Figures 4a, S3). In addition, deletion of other sequences from EBEs in rice also conferred disease resistance (Blanvillain‐Baufume *et al*., 2017; Li *et al*., 2012), suggesting that the PthA4 EBE of *CsLOB1* may contain other *cis*‐elements that affect citrus canker resistance, which were not detected in the present study.

The S2 sgRNA more efficiently mediated Cas9 nuclease to delete the TATA box from the *CsLOB1* promoter compared with the other four sgRNAs tested. Cas9 nuclease cuts specifically between the third and fourth nucleotides upstream of the PAM (Anders *et al*., 2014), whereas the cut site mediated by S2 sgRNA was between the second and third bases in the TATA box (Figure 2a). This is likely to be an important factor in the efficacy of S2 sgRNA. The result provides useful information for the design of efficient sgRNAs to modify the EBE of susceptibility genes, which overlap or encompass the TATA box, for improvement of plant disease resistance. Meanwhile, it also suggests that the cut site should be placed closer to or in targeted bases or elements to efficiently edit them in CRISPR/Cas9‐mediated genome modification.

In summary, we demonstrated that promoter editing of the disease‐susceptibility gene *CsLOB1* in Wanjincheng orange confers resistance to citrus canker. All mutant plants were morphologically normal compared with the wild type (Figure S9), indicating that modification of the *CsLOB1* promoter does not disrupt plant development. Further research is in progress to dissect the different resistance mechanisms among the mutant lines obtained, and to edit other economically important citrus cultivars using the constructs with S2 sgRNA, which showed high efficacy. In addition, the disease resistance of transgenic plants observed under controlled conditions must be confirmed in field trials.

## Materials and methods

### Plant and pathogen materials

Wanjincheng orange, Chandler pummelo and Satsuma mandarin plants were obtained from the National Citrus Germplasm Repository, Chongqing, China. All wild‐type and transgenic plants were grown in a greenhouse maintained at 28 °C.

A type A strain of *Xcc*,* Xcc*YN1, was isolated from naturally infected sweet orange leaves from an orchard in Yunnan province, China. Preparation of the bacterial suspensions for infection experiments was performed as described by Zou *et al*. (2014).

### High‐resolution melting analysis

Genomic DNA was extracted from citrus leaves using the Plant DNeasy Prep Kit (Qiagen, Beijing, China). To investigate the genotypes of the *CsLOB1* promoter in Wanjincheng orange, the primer pair Hp171‐1f/Hp171‐1R (Table S6) was designed on the basis of the indel of adenine (G) located just downstream of the PthA4 EBE in the *CsLOB1* promoter (Hu *et al*., 2014; Li *et al*., 2014). The PCR were performed in a final volume of 10 μL, containing 5 μL Precision Melt Supermix for HRM analysis (Bio‐Rad #172–5112), 0.5 μL of each primer (10 μm) and 2 μL genomic DNA. The PCR protocol was 95 °C for 5 min, then 30 cycles of 94 °C for 30 s, 56 °C for 30 s and 72 °C for 30 s, followed by 94 °C for 30 s and 25 °C for 60 s. Using Chandler pummelo and Satsuma mandarin as controls, indels in the amplified PCR products were analysed using a LightScanner^®^ 96 Hi‐Res Melting^®^ system (Idaho Technology, Salt Lake City, UT). Amplicons were subjected to direct sequencing using the Hp171‐1R primer. The experiment was repeated three times.

### Vector construction

The pCas9‐GN vector (Figure S10) was used to construct CRISPR/Cas9 expression vectors for citrus transformation. To target the PthA4 EBE region in the *CsLOB1* promoter, a series of paired DNA oligos (Table S6) were synthesized by Invitrogen Biotech (Shanghai, China). The synthesized oligos were annealed and inserted into the *Bbs*I sites of the pUC119‐gRNA vector in accordance with the manufacturer's protocol (Cong *et al*., 2013). Expression of sgRNAs was driven by the AtU6‐1 promoter from *Arabidopsis thaliana* L. Subsequently, the sgRNA expression cassette containing a specific target site was unloaded with *Bam*HI/*Sal*I and inserted into the *Bam*HI/*Sal*I‐digested pCas9‐GN vector to generate the pCas9/CsLOB1sgRNA plant expression vectors. Five plasmids, each containing a different sgRNA, were constructed (Figure 2b).

### Citrus transformation

The Cas9/sgRNA expression binary vectors were transformed into *Agrobacterium tumefaciens* strain EHA105 by electroporation. *Agrobacterium*‐mediated transformation of Wanjincheng orange epicotyl explants was performed as previously described (Peng *et al*., 2015). Kanamycin‐resistant shoots were first detected by GUS histochemical staining to identify transgenic plants (Zou *et al*., 2008). The GUS‐positive shoots were grafted onto Troyer citrange [*Poncirus trifoliata* (L.) Raf. × *C. sinensis*] seedlings *in vitro*. Integration and expression of the *pcoCas9* gene in transformed shoots were further confirmed by PCR analysis. The recovered shoots were further grafted onto Troyer citrange seedlings in the greenhouse.

### Sequencing analysis

All transgenic plants as well as the wild type were subjected to PCR using the gene‐specific primer pair LOBp‐f/LOBp‐r (Table S6) to amplify DNA fragments across the target sites. The PCR amplicons were cloned into the pGEM^®^‐T Easy vector (Promega, Madison, WI) for Sanger sequencing. A minimum of 10 clones per transgenic plant were sequenced. For direct sequencing, the amplified products were purified and subjected to sequencing using the primer pair seq‐f/seq‐r (Table S6). The sequence chromatograms were analysed with Vector NTI and SnapGene Viewer software.

### Gene expression analysis

RNA was extracted using the EASYspin Plant RNA Extraction Kit following the manufacturer's instructions (Aidlab, Beijing, China). RNA (1 μg) was reverse transcribed into cDNA using the iScript™ cDNA Synthesis Kit (Bio‐Rad, Hercules, CA). Detection of gene expression was performed by qPCR using the 2× iQ™ SYBR^®^ Green Supermix (Bio‐Rad). The primers used are listed in Table S6. The PCR were carried out as follows: a pretreatment (95 °C for 5 min) followed by 40 amplification cycles (95 °C for 20 s; 60 °C for 60 s). Experiments were repeated three times. Using the citrus *Actin* gene (GenBank accession no. GU911361.1) for normalization, the relative expression levels were calculated as described by Zou *et al*. (2014).

### Assay of resistance to citrus canker

The *in vitro* assay for disease resistance of mutant plants to *Xanthomonas citri subsp. citri* was performed as described by Peng *et al*. (2015). Fully mature healthy leaves (about 3 months old) were inoculated with *Xcc*YN1. Four leaves per line were tested. Six infected sites, each comprising six punctures, per leaf were made with a pin (0.5 mm in diameter). 1 μL bacterial suspension (1 × 10^5^ CFU/mL) was applied to each puncture site. Photographs were taken at 5 and 9 dpi. The area of all diseased spots was assessed with ImageJ 2.0 software (National Institutes of Health, Bethesda, MD). The disease intensity of an individual line was based on 36 punctures in four leaves using the following rating index: 0, <0.25 mm^2^ (the size of the inoculating pin); 1, 0.25–0.75 mm^2^; 3, 0.75–1.25 mm^2^; 5, 1.25–1.75 mm^2^; 7, >1.75 mm^2^. The disease index (DI), indicating the level of resistance to *Xcc*, was calculated with the formula:

DI=∑[no. of each index×the corresponding index]/(36×7)×100.The experiment was repeated six times.

Growth of *Xcc* in mutant plants was performed as described by Peng *et al*. (2015). At 0, 1, 3, 5, 7 and 9 dpi, bacterial colonies were counted and used to estimate the number of bacterial cells per unit area (cm^2^) of leaf.

The resistance of mutant plants was further determined by *in vivo* infiltration (Abe and Benedetti, 2016). Leaves (about 3 months old) were infiltrated with *Xcc*YN1 bacterial suspensions (1 × 10^5^ CFU/mL). The inoculated plants were monitored daily for appearance of canker symptoms.

### Analysis of potential off‐target sequences

The Cas9/sgRNA analysis software (http://cbi.hzau.edu.cn/cgi-bin/CRISPR) was used to analyse potential off‐target sequences of Cas9/sgRNA. On the basis of these sequences, primers were designed to analyse the potential off‐target fragments (Table S7). The PCR products were cloned into the pGEM^®^‐T Easy vector for sequencing. The sequence chromatograms were analysed with SnapGene Viewer software.

## Author contribution statement

X. Zou designed the experiments, analysed the data and wrote the manuscript. A. Peng performed citrus genetic transformation, sequencing analysis and resistance evaluation. S. Chen analysed the data. T. Lei and L. Wu performed HRM and off‐target analyses. L. Xu performed PCR and GUS analysis. Y. He constructed the vectors. L. Yao performed resistance evaluation. All of the authors read and approved the manuscript.

## Conflict of interest

The authors declare that they have no conflict of interest.

## Supporting information


**Figure S1** Sequences of alleles of the *CsLOB1* promoter in Wanjincheng orange (*Citrus sinensis* Osbeck).
**Figure S2** Molecular confirmation of transgenic plants.
**Figure S3** Efficient targeted gene editing using CRISPR/Cas9 in Wanjincheng orange (*Citrus sinensis* Osbeck).
**Figure S4** Chromatogram (a) and sequence (b) characteristics of the S2‐5 mutation line of Wanjincheng orange (*Citrus sinensis* Osbeck).
**Figure S5** Expression of *CsLOB1* in citrus mutants after *Xanthomonas citri* subsp. *citri* (*Xcc*) inoculation.
**Figure S6** Coding sequences of *CsLOB1* ^*G*^ and *CsLOB1* ^*−*^ and the corresponding amino acid sequences of Wanjincheng orange (*Citrus sinensis* Osbeck).
**Figure S7** Citrus canker symptoms on leaves of Wanjincheng orange (*Citrus sinensis* Osbeck) mutants.
**Figure S8** Disease resistance in transgenic plants of Wanjincheng orange (*Citrus sinensis* Osbeck).
**Figure S9** One‐year‐old modified plants growing in the greenhouse.
**Figure S10** T‐DNA structure of the PCas9‐GN plasmid used in the study.
**Table S1** Genetic analysis of the *CsLOB1* promoter in Wanjincheng orange (*Citrus sinensis* Osbeck).
**Table S2** Statistical analysis of transgenic lines of Wanjincheng orange (*Citrus sinensis* Osbeck) with mutations induced by five sgRNAs.
**Table S3** Characteristics of indels in transgenic plants of Wanjincheng orange (*Citrus sinensis* Osbeck).
**Table S4** Mutation frequency in the effector binding element (EBE) in transgenic plants of Wanjincheng orange (*Citrus sinensis* Osbeck).
**Table S5** Putative CRISPR/Cas9 off‐target sites.
**Table S6** Primers used in the study.
**Table S7** Primers used to investigate CRISPR/Cas9‐mediated off‐targeting.
