# Supplementary material for: Engineering canker‐resistant plants through CRISPR/Cas9‐targeted editing of the susceptibility gene CsLOB1 promoter in citrus
Source: Plant Biotechnol J. 2017 May 3;15(12):1509–19. doi: 10.1111/pbi.12733 (PMC5698050; doi:10.1111/pbi.12733)
Supplement: Supplementary file 1 — Figure S1 Sequences of alleles of the CsLOB1 promoter in Wanjincheng orange (Citrus sinensis Osbeck). Figure S2 Molecular confirmation of transgenic plants. Figure S3 Efficient targeted gene editing using CRISPR/Cas9 in Wanjincheng orange (Citrus sinensis Osbeck). Figure S4 Chromatogram (a) and sequence (b) characteristics of the S2‐5 mutation line of Wanjincheng orange (Citrus sinensis Osbeck). Figure S5 Expression of CsLOB1 in citrus mutants after Xanthomonas citri subsp. citri (Xcc) inoculation. Figure S6 Coding sequences of CsLOB1 G and CsLOB1 − and the corresponding amino acid sequences of Wanjincheng orange (Citrus sinensis Osbeck). Figure S7 Citrus canker symptoms on leaves of Wanjincheng orange (Citrus sinensis Osbeck) mutants. Figure S8 Disease resistance in transgenic plants of Wanjincheng orange (Citrus sinensis Osbeck). Figure S9 One‐year‐old modified plants growing in the greenhouse. Figure S10 T‐DNA structure of the PCas9‐GN plasmid used in the study. Table S1 Genetic analysis of the CsLOB1 promoter in Wanjincheng orange (Citrus sinensis Osbeck). Table S2 Statistical analysis of transgenic lines of Wanjincheng orange (Citrus sinensis Osbeck) with mutations induced by five sgRNAs. Table S3 Characteristics of indels in transgenic plants of Wanjincheng orange (Citrus sinensis Osbeck). Table S4 Mutation frequency in the effector binding element (EBE) in transgenic plants of Wanjincheng orange (Citrus sinensis Osbeck). Table S5 Putative CRISPR/Cas9 off‐target sites. Table S6 Primers used in the study. Table S7 Primers used to investigate CRISPR/Cas9‐mediated off‐targeting. [file PBI-15-1509-s001.doc]

Engineering canker-resistant plants through CRISPR/Cas9-targeted editing of the susceptibility gene *CsLOB1* promoter in citrus

Aihong Peng^1,2^,^*^, Shanchun Chen^1,2,*^, Tiangang Lei^1,2^, Lanzhen Xu^1,2^, Yongrui He^1,2^, Liu Wu^2^, Lixiao Yao^1,2^ and Xiuping Zou^1,2,†^

^1^Citrus Research Institute, Chinese Academy of Agricultural Sciences and National Center for Citrus Variety Improvement, Chongqing 400712, P. R. China

^2^Citrus Research Institute, Southwestern University, Chongqing 400716, P. R. China­­

^†^Corresponding author

Tel: 86-23-68349019

Fax: 86-23-68349020

E-mail: [zouxiuping@cric.cn](mailto:zouxiuping@cric.cn)

^*^Aihong Peng and Shanchun Chen contributed equally to this work.

**Table S1.** Genetic analysis of the *CsLOB1* promoter in Wanjincheng orange (*Citrus sinensis* Osbeck)

| ^a^Test | No. of Total clones | No. of *CsLOB1^G^* | No. of *CsLOB1*^–^ | | ^b^χ^2^ | | --- | |
| --- | --- | --- | --- | --- | --- |
| T1 | 41 | 37 | 4 | 1.08 |
| T2 | 45 | 35 | 10 | 0.02 |
| T3 | 36 | 29 | 7 | 0.08 |

^a^ Each test was performed as follows: isolation of leaf DNA, PCR amplification, T-cloning and sequencing.

^b^$\chi_{C}^{2}=\sum\frac{(|O-E|-1/2)^{2}}{E}$(*O,* observed frequency; *E,* expected frequency): $\chi_{0.05,1}^{2}$=3.84. The values of χ^2^ reflect the goodness-of-fit of *CsLOB1^G^*:*CsLOB1*^–^ to a 3:1 ratio.

**Table S2.** Statistical analysis of transgenic lines of Wanjincheng orange (*Citrus sinensis* Osbeck) with mutations induced by five sgRNAs

| sgRNA | No. of transgenic lines | No. of lines with mutation (%)^a^ | No. of lines with the EBE mutation (%)^b^ |
| --- | --- | --- | --- |
| S1 | 26 | 3 (11.5) | 1 (33.3) |
| S2 | 32 | 10 (31.3) | 4 (40.0) |
| S3 | 24 | 8 (33.3) | 3 (37.5) |
| S4 | 17 | 11 (64.7) | 5 (35.5) |
| S5 | 11 | 6 (54.5) | 3 (50.0) |
| Total | 110 | 38 (34.5) | 16 (42.1) |

Ten clones per line were sequenced.

^a^ Based on the number of mutant plants out of the total number of transgenic plants tested.

^b^ Based on the number of plants with mutations in the EBE out of the total number of mutant plants tested.

**Table S3.** Characteristics of indels in transgenic plants of Wanjincheng orange (*Citrus sinensis* Osbeck)

| line | Genotype^a^ | Mutation type ^b^ | *CsLOB1^G^* mutation | *CsLOB1*^–^ mutation | EBE  mutation |
| --- | --- | --- | --- | --- | --- |
| S1-3 | Chimera | G(i1s2), G(wt), –(wt) | Y* | N* | Y* |
| S1-12 | Chimera | G(s1), G(wt), –(wt) | Y | N | N |
| S1-13 | Heterozygote | G(d2), –(wt) | Y | N | N |
| S2-2 | Chimera | G(s1), G(s4), –(wt) | Y | N | N |
| S2-3 | Homozygote | G(d2), –(d2) | Y | Y | N |
| S2-4 | Chimera | G(s1), G(wt), –(wt) | Y | N | N |
| S2-5 | Bi-allele | G(d182), –(d2) | Y | Y | N |
| S2-6 | Homozygote | G(d31), –(d31) | Y | Y | Y |
| S2-8 | Chimera | G(i1), G(d31), G(d36), G(d5), –(i1) | Y | Y | Y |
| S2-9 | Chimera | G(d4), G(d37) –(i1) | Y | Y | Y |
| S2-12 | Biallele | G(d14), –(i1) | Y | Y | Y |
| S2-14 | Chimera | G(d2), G(d4), G(d31), G(wt), –(wt) | Y | N | N |
| S2-18 | Chimera | G(i1), G(i1), G(d1), G(wt), –(wt) | Y | N | N |
| S3-3 | Chimera | G(d6), G(s1), G(wt), –(wt) | Y | N | Y |
| S3-4 | Chimera | G(d4), G(i1), –(wt) | Y | N | N |
| S3-5 | Chimera | G(i1d2), G(d24), G(wt), –(wt) | Y | N | Y |
| S3-6 | Chimera | G(d4), G(i1),–(i1), –(wt) | Y | Y | N |
| S3-7 | Chimera | G(d1), G(d4), G(d4), G(i1), –(wt) | Y | N | N |
| S3-8 | Chimera | G(d8), G(d3), G(d4s2), –(wt) | Y | N | N |
| S3-9 | Chimera | G(d5), G(i1), G(s1), G(wt), –(wt) | Y | N | Y |
| S3-11 | Chimera | G(i2), G(wt), –(wt) | Y | N | N |
| S4-1 | Chimera | G(d4), G(d4s1), G(wt), –(wt) | Y | N | N |
| S4-2 | Chimera | G(s1), G(wt), –(wt) | Y | N | Y |
| S4-4 | Chimera | G(d5), G(d1), G(wt), –(wt) | Y | N | N |
| S4-5 | Heterozygote | G(d4), –(wt) | Y | N | N |
| S4-6 | Heterozygote | G(d4), –(wt) | Y | N | N |
| S4-7 | Heterozygote | G(d1), –(wt) | Y | N | N |
| S4-8 | Chimera | G(d5), G(s1), –( d4) | Y | Y | Y |
| S4-10 | Chimera | G(s1), G(wt), –(d5) | Y | Y | N |
| S4-11 | Chimera | G(d6), G(i1), G(i2), –(wt) | Y | N | Y |
| S4-13 | Heterozygote | G(i1), –(wt) | Y | N | Y |
| S4-15 | Heterozygote | G(d4s1), –(wt) | Y | N | Y |
| S5-1 | Chimera | G(i1), G(wt), –(d3) | Y | Y | N |
| S5-2 | Chimera | G(d13), G(d4s1), G(s1), G(d2), G(d1), G(wt), –(wt) | Y | N | N |
| S5-3 | Chimera | G(d43), G(wt), –(d11), –(wt) | Y | Y | Y |
| S5-4 | Chimera | G(d3), G(wt), –(wt) | Y | N | N |
| S5-13 | Chimera | G(d48), G(d5), G(wt), –(d38), –(wt) | Y | Y | Y |
| S5-15 | Chimera | G(s2), G(wt), –(wt) | Y | N | Y |

^a^ Chimera refers to a plant with at least three distinct alleles detected at the target site.

^b^ “G()” and “–()” indicate that the mutation was in *CsLOB1^G^* and *CsLOB1^–^*, respectively. In parentheses, “d#”, “i#” and “s#” indicate the number of nucleotides deleted, inserted and substituted at the target site, respectively.

* “N” and “Y” indicate “No” and “Yes”, respectively.

**Table S4.** Mutation frequency in the effector binding element (EBE) in transgenic plants of Wanjincheng orange (*Citrus sinensis* Osbeck)

| Line | Clones sequenced (*CsLOB1^G^*/*CsLOB1*^–^) | *CsLOB1^G^* mutants (%)^a^ | *CsLOB1*^–^ mutants (%) ^b^ | *CsLOB1^G^*mutants with EBE mutation (%) ^c^ | *CsLOB1*^–^ mutants with EBE mutation (%) ^d^ |
| --- | --- | --- | --- | --- | --- |
| S1-3 | 32/8 | 4 (12.5) | 0 (0.0) | 4 (50.0) | 0 (0.0) |
| S2-5* | 58/11 | 58 (100.0) | 11 (100.0) | 0 (0.0) | 0 (0.0) |
| S2-6 | 65/10 | 65 (100.0) | 10 (100.0) | 65 (100.0) | 10 (100.0) |
| S2-8 | 37/3 | 37 (100.0) | 3 (100.0) | 4 (10.8) | 0 (0.0) |
| S2-9 | 35/3 | 35 (100.0) | 3 (100.0) | 5 (14.3) | 0 (0.0) |
| S2-12 | 43/7 | 43 (100.0) | 7 (100.0) | 43 (100.0) | 0 (0.0) |
| S2-14 | ND | ND | ND | ND | ND |
| S3-3 | 34/6 | 8 (23.6) | 0 (0.0) | 4 (11.8) | 0 (0.0) |
| S3-5 | 34/6 | 6 (17.6) | 0 (0.0) | 3 (8.8) | 0 (0.0) |
| S3-9 | 34/6 | 20 (58.8) | 0 (0.0) | 5 (14.7) | 0 (0.0) |
| S4-2 | 32/8 | 8 (25.0) | 0 (0.0) | 8 (25.0) | 0 (0.0) |
| S4-8 | 31/9 | 31 (100.0) | 9 (100.0) | 4 (12.9) | 0 (0.0) |
| S4-11 | 39/4 | 39 (100.0) | 0 (0.0) | 39 (100.0) | 0/4 (0.0) |
| S4-13 | 31/6 | 31 (100.0) | 0 (0.0) | 31 (100.0) | 0 (0.0) |
| S4-15 | ND | ND | ND | ND | ND |
| S5-2 | ND | ND | ND | ND | ND |
| S5-3 | 29/4 | 7 (24.1) | 2 (50.0) | 7 (17.2) | 2 (50.0) |
| S5-13 | 54/20 | 24 (44.4) | 10 (50.0) | 14 (18.5) | 10 (50.0) |
| S5-15 | ND | ND | ND | ND | ND |

^a^ Based on the number of *CsLOB1^G^* mutant clones out of the total number of *CsLOB1^G^* clones sequenced.

^b^ Based on the number of *CsLOB1*^–^ mutant clones out of the total number of *CsLOB1*^–^ clones sequenced.

^c^ Based on the number of *CsLOB1^G^* mutant clones with EBE mutations out of the total number of *CsLOB1^G^* clones sequenced.

^d^ Based on the number of *CsLOB1*^–^ mutant clones with EBE mutations out of the total number of *CsLOB1*^–^ clones sequenced

ND: no detection

**Table S5.** Putative CRISPR/Cas9 off-target sites

| Name of putative off-target locus | Putative off-target locus | Sequence of the putative off-target site | No. of mismatching bases |
| --- | --- | --- | --- |
| O1 | chr7:+24487268 | **A**TTT**T**TATA**T**AGAAAGGAAA**AAG** | 3 |
|
|
|
| O2 | chrUn:-18976355 | GTTTATAT**T**GAGAAAGGAA**CAGG** | 2 |
|
|
|
| O3 | chr7:+10471336 | **A**TTTATAT**CC**AGAAAGGAAA**AAG** | 3 |
|
|
|
| O4 | chrUn:+33253381 | **A**TTT**T**TATAGA**A**AAAGGAAA**TAG** | 3 |
|
|
|
| O5 | chr1:-3586814 | G**C**T**A**ATA**A**A**A**AGAAAGGAAA**AAG** | 4 |
| O6 | chrUn:+64516242 | **T**TTTATATA**T**AGAAAG**C**AAA**CGG** | 3 |
|
|
|
| O7 | chr1:+14960328 | GTTTATATAGA**A**AAAG**A**AAA**AAG** | 2 |
|
|
|
| O8 | chr8:-5830114 | **C**TTTATATA**A**AG**T**AAGGAAA**AAG** | 3 |
|
|
|
| O9 | chr4:+9246320 | **AA**TTA**G**A**G**AGAGAAAGGAAA**AAG** | 4 |
|
|
|
| O10 | chr7:+4175356 | **T**TTT**T**TATAGAGAAAGGA**T**A**AAG** | 3 |
|
|
|
| O11 | chrUn:-33809320 | **T**TTTAT**T**TAGAGAAAG**T**AAA**AGG** | 3 |
|

The protospacer adjacent motif (PAM) sequence (NGG or the analogue NAG) is indicated in bold. Mismatch nucleotides are marked in red

**Table S6.** Primers used in the study

| name | sequence（5′→3′） | Restriction  site | Usage |
| --- | --- | --- | --- |
| HP171-1f | CATTCTTGCCTTTTCCTTTCTCTA | *-* | HRM analysis of the CsLOB1 type in citrus |
| Hp171-1R | GTAAGGGATGAGGAGGAGCTG | *-* |
| 35S-f | cg*AAGCTT*CCATGGAGTCAAAGATTCAAATAG | *Hin*dIII | PCR the CaMV35S promoter |
| 35S-r | tc*AGATCT*TACCCTGTCCTCTCCAAATGAAATG | *Bgl*II |
| LOBp-f | AC*AAGCTT*CACATATTTGAAAGTACATCCATAAC | *Hin*dIII | Clone CsLOB1 promoter |
| LOBp-r | AT*GGATCC*TTTTGAGAGAAGAAAACTGT | *Bam*HI |
| S1-f | *GATTG*AGAGATATATTTGGGGAAAA | *Bbs*I | Construct CsLOBp:sgRNA1 |
| S1-r | *AAAC*TTTTCCCCAAATATATCTCTC | *Bbs*I |
| S2-f | *GATTG*GAAAAGGAAAGAGATATATT | *Bbs*I | Construct CsLOBp:sgRNA2 |
| S2-r | *AAAC*AATATATCTCTTTCCTTTTCC | *Bbs*I |
| S3-f | *GATTG*AAAGGGGTTTATATAGAGAA | *Bbs*I | Construct CsLOBp:sgRNA3 |
| S3-r | *AAAC*TTCTCTATATAAACCCCTTTC | *Bbs*I |
| S4-f | *GATTG*GAAACAAAGTTCAAGGCAAA | *Bbs*I | Construct CsLOBp:sgRNA4 |
| S4-r | *AAAC*TTTGCCTTGAACTTTGTTTC | *Bbs*I |
| S5-f | *GATTG*GTTTATATAGAGAAAGGAAA | *Bbs*I | Construct CsLOBp:sgRNA5 |
| S5-r | *AAAC*TTTCCTTTCTCTATATAAAC | *Bbs*I |
| cas9-f | AGAAGGACCTCATCATCAAGC | - | PCR transgene integration in citrus |
| cas9-r | GATGAGAGTAGCGTCGAGAACC | - |
| LOB-f | atggaatgcaaacacaaaattaatg | - | Clone the cDNAs of *CsLOB1^G^* and *CsLOB1^–^* |
| LOB-r | tcatgtccacagaggctccca | - |
| Seq-f | CGTCATTCAATTAAAATTAATGAC | *-* | Direct sequence target editing |
| Seq-r | agtgagaaagcaaagacagtaag | *-* |
| qcas9-f | CCCTTACCAACCTTGGTGCT | *-* | Expression analysis of the *pcoCas9* in transgenic plants |
| qcas9-r | TGTCCAGCCTTCTTGGTAGC | *-* |
| qLOB-f | CTGCCAGAATCTCAACGAGC | - | Expression analysis of the *CsLOB1* |
| qLOB-r | TTGGCTAACTGAGCCTGAAGC | - |
| qACT-f | CATCCCTCAGCACCTTCC | - | Expression analysis of citrus *actin* |
| qACT-r | CCAACCTTAGCACTTCTCC | - |

Note: The sequences of restriction enzyme sites are in italics.

**Table S7.** Primers used to investigate CRISPR/Cas9-mediated off-targeting

| Primer name | Sequence (5′→3′) | putative off-target locus amplified |
| --- | --- | --- |
| O1-f | TTATAGGAAAGTAGGAATGTAGAGC | chr7:+24487268 |
| O1-r | GAAATGAATACGGCCTATTA |
| O2-f | TTCTTCATGTCCATTTAGAG | chrUn:-18976355 |
| O2-r | AGGCACACAAAGTAGCTTTT |
| O3-f | GCCACCTCAGCTTCACTAAT | chr7:+10471336 |
| O3-r | GGCGTTGGAGAATGAAGACC |
| O4-f | GAAAAGACCAAAAAAGCCATCTTCT | chrUn:+33253381 |
| O4-r | CCAGTTTAATTCCAATGTTGGATAG |
| O5-f | GTGCAATGCCAAAGACGAGA | chr1:-3586814 |
| O5-r | GGGCTTATACTGGCAAGCCT |
| O6-f | TCCACTCTTGCACTCCATTGT | chrUn:+64516242 |
| O6-r | GTGATCTGGATTTGGCAGCATT |
| O7-f | TCCCCATCTACAATCACATGGT | chr1:+14960328 |
| O7-r | ACACTCGAGCGATGTGGAAT |
| O8-f | TCATCCACCATTGAACTACATGA | chr8:-5830114 |
| O8-r | GGAAGGGACATCAACACCCA |
| O9-f | CCAACATATGCCCACCTCCA | chr4:+9246320 |
| O9-r | TGAGCGAATTCACGTACACA |
| O10-f | TGATTGGACGAGACAACTTCCA | chr7:+4175356 |
| O10-r | AACACCCAATCAATCCAATCCT |
| O11-f | ACCTGTCGGTCTTTCAACGG | chrUn:-33809320 |
| O11-r | GTGTGCAGTGCAACAAGTCA |


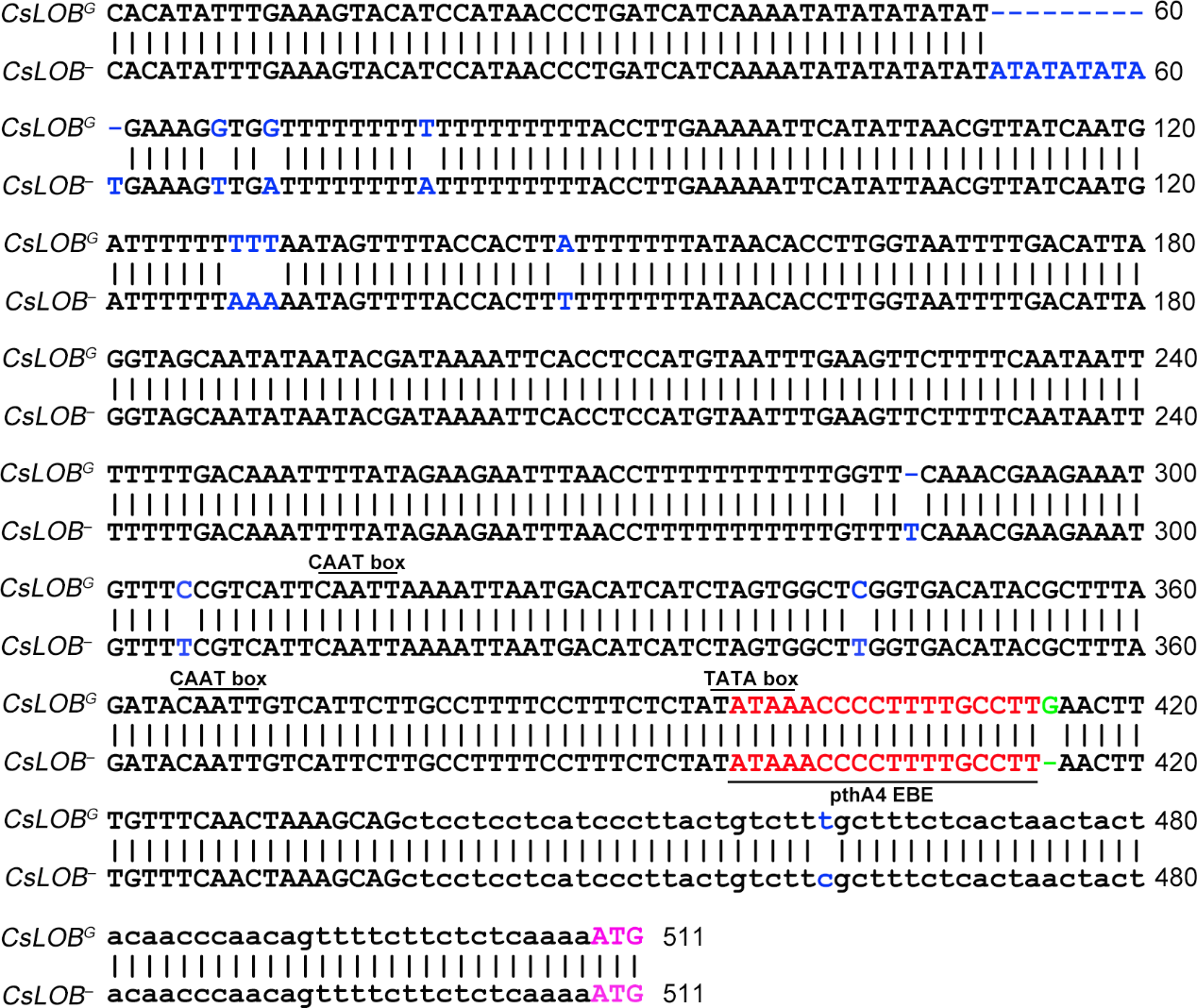


**Figure S1.** Sequences of alleles of the *CsLOB1* promoter in Wanjincheng orange (*Citrus sinensis* Osbeck). Red letters indicate the effector binding element (EBE) of *Xanthomonas axonopodis* pv*. citri*. Green letters indicate the G indel that distinguishes the alleles. Blue letters indicate other indels differing in the promoter between *CsLOB1^G^* and *CsLOB1*^–^. Purple letters indicate the start codon.


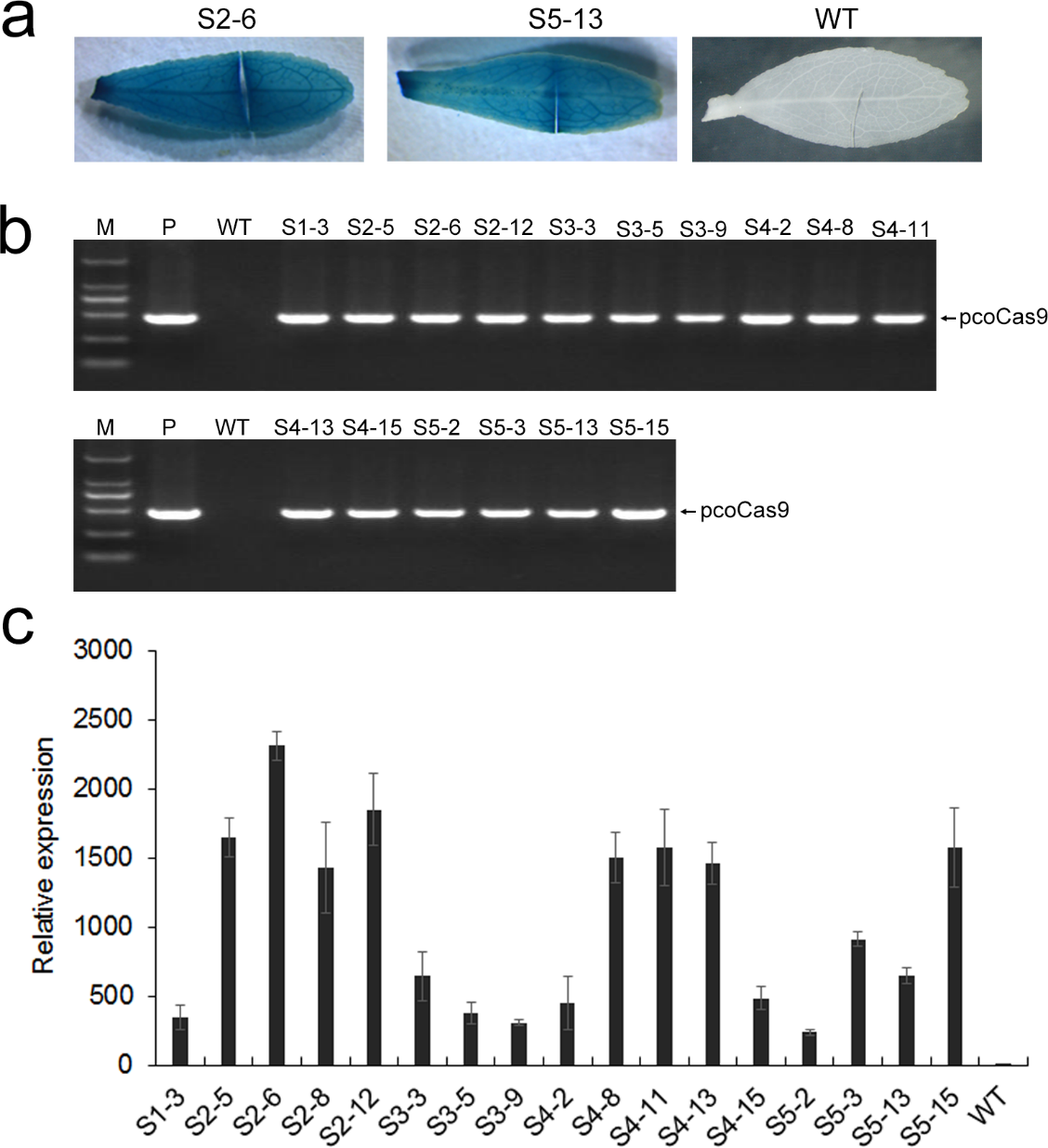


**Figure S2.** Molecular confirmation of transgenic plants. (a**)** Kanamycin-resistant shoots were used to investigate the integration of *gus* by GUS histochemical staining. Blue staining was detected in transgenic leaves, whereas no staining was observed in the wild type. (b) Integration of transgenes in transgenic lines was confirmed by PCR. The primers cas9-f/cas9-r were used to amplify *pcoCas9* in GUS-positive transgenic shoots. Bands of approximately 500 bp were detected in transgenic shoots. (**c)** Quantitative real-time PCR analysis of *pcocas9* expression in the representative transgenic lines. *pcoCas9* transcripts in leaf tissue of transgenic lines were investigated by PCR. Relative expression levels were calculated by comparing *pcoCas9* expression in transgenic lines with that in the wild type.


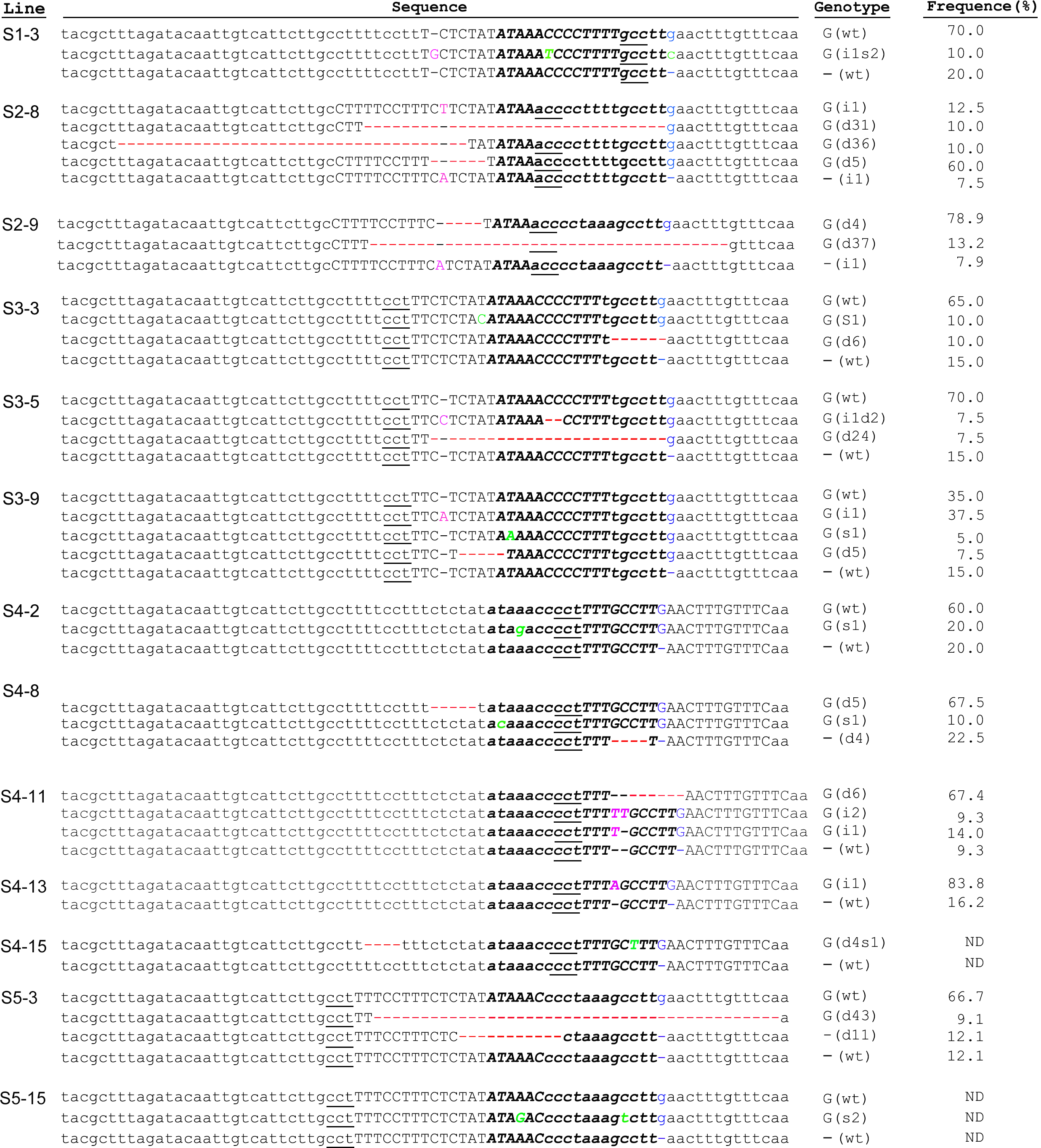


**Figure S3.** Efficient targeted gene editing using CRISPR/Cas9 in Wanjincheng orange (*Citrus sinensis* Osbeck). The effector binding element is in italics. The sgRNAs are in upper-case letters and the protospacer adjacent motif sites (PAM) are underlined. The indel distinguishing the promoter of *CsLOB1^G^* and *CsLOB1*^–^ is in blue. Red dashes indicate deleted nucleotides. Pink letters indicate inserted nucleotides. Green letters indicate substituted nucleotides. “G ()” and “– ()” indicate mutations in *CsLOB1^G^* and *CsLOB1*^–^, respectively. In parentheses, “d#”, “i#”, and “s#” indicate the number of nucleotides deleted, inserted and substituted at the target site, respectively. WT and wt, wild type. Frequency (%) was calculated based on the number of clones with the same mutation out of the total number of clones sequenced. Forty clones per line were sequence to investigate mutations.


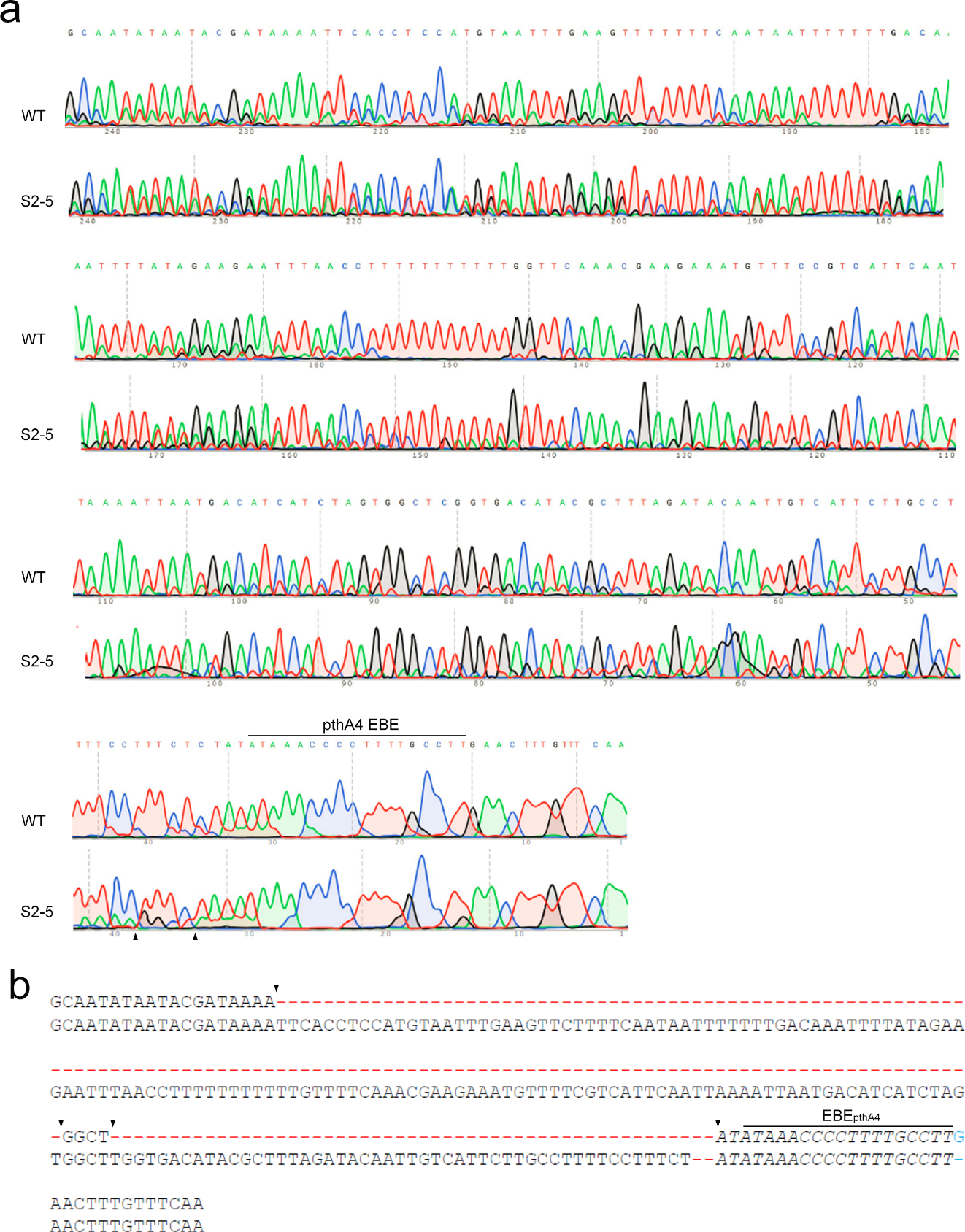


**Figure S4.** Chromatogram (a) and sequence (b) characteristics of the S2-5 mutation line of Wanjincheng orange (*Citrus sinensis* Osbeck). Arrowheads indicate deletion of loci. The indel distinguishing the promoter of *CsLOB1^G^* and *CsLOB1*^–^ is highlighted in blue. Red dashes indicate deleted nucleotides.


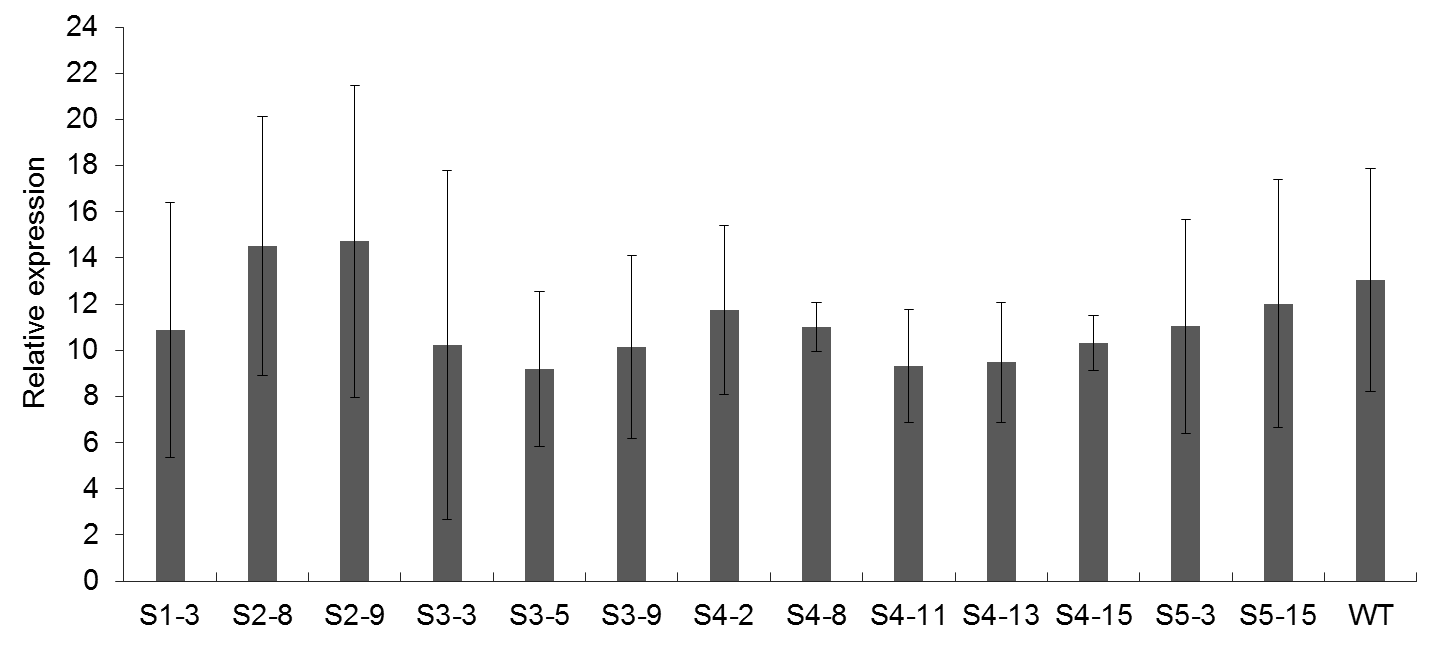


**Figure S5.** Expression of *CsLOB1* in citrus mutants after *Xanthomonas citri* subsp. *citri* (*Xcc*) inoculation. After one day of *Xcc* infection, *CsLOB1* transcripts in leaves were analyzed by quantitative real-time PCR. Relative expression levels of *CsLOB1* were determined by comparing the *CsLOB1* transcript levels after *Xcc* inoculation with that after water inoculation. Error bars indicate the standard deviation from three independent tests.


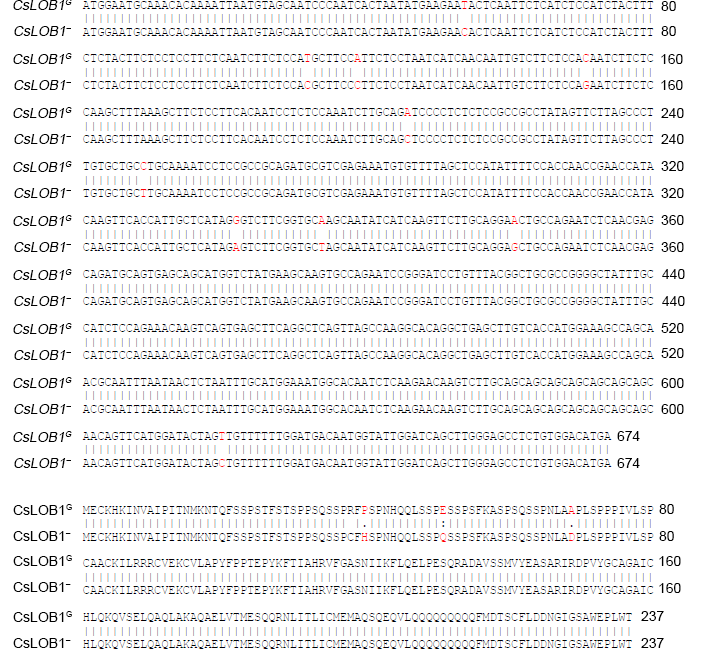


**Figure S6.** Coding sequences of *CsLOB1^G^* and *CsLOB1^–^* and the corresponding amino acid sequences of Wanjincheng orange (*Citrus sinensis* Osbeck). Red letters indicate differences in sequence. *CsLOB1* cDNA from Wanjincheng orange leaves were amplified using LOB-f/ LOB-r primers (Table S7), cloned into the pGEM®-T Easy vector, and sequenced. The sequences were determined according to data for 10 clones.


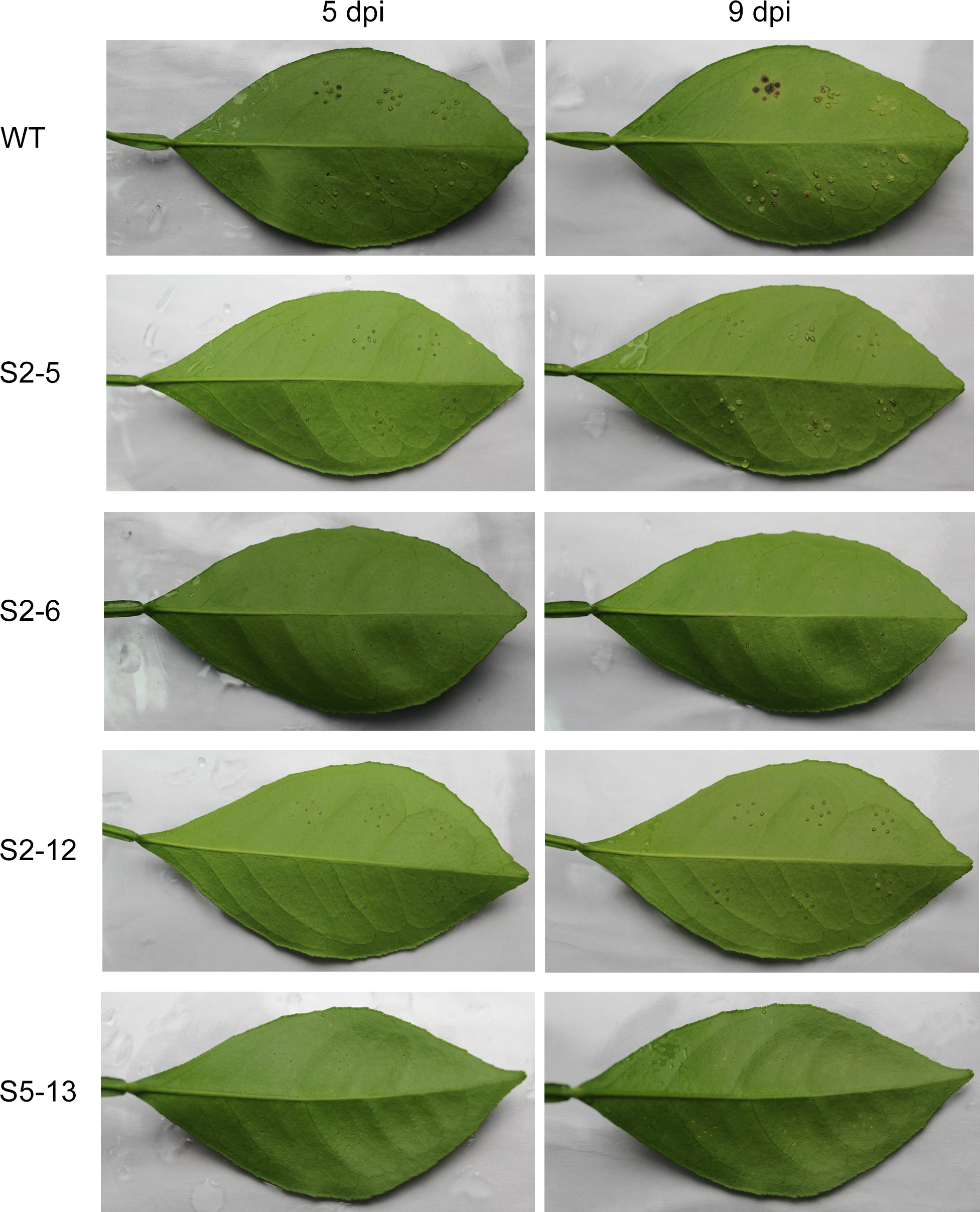


**Figure S7.** Citrus canker symptoms on leaves of Wanjincheng orange (*Citrus sinensis* Osbeck) mutants. Photographs were recorded 5 and 9 days after *Xanthomonas citri* subsp. *citri* inoculation. WT, wild type; dpi, days post-inoculation.


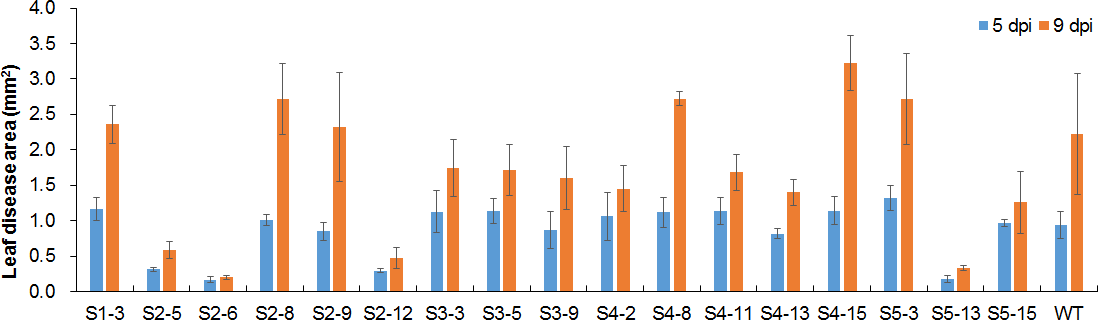


**Figure S8.** Disease resistance in transgenic plants of Wanjincheng orange (*Citrus sinensis* Osbeck). Leaf disease area (mm^2^) caused by *Xanthomonas citri* subsp. *citri* infection were determined at 5 and 9 days post-inoculation (dpi). Error bars indicate SD. The experiment was repeated three times.


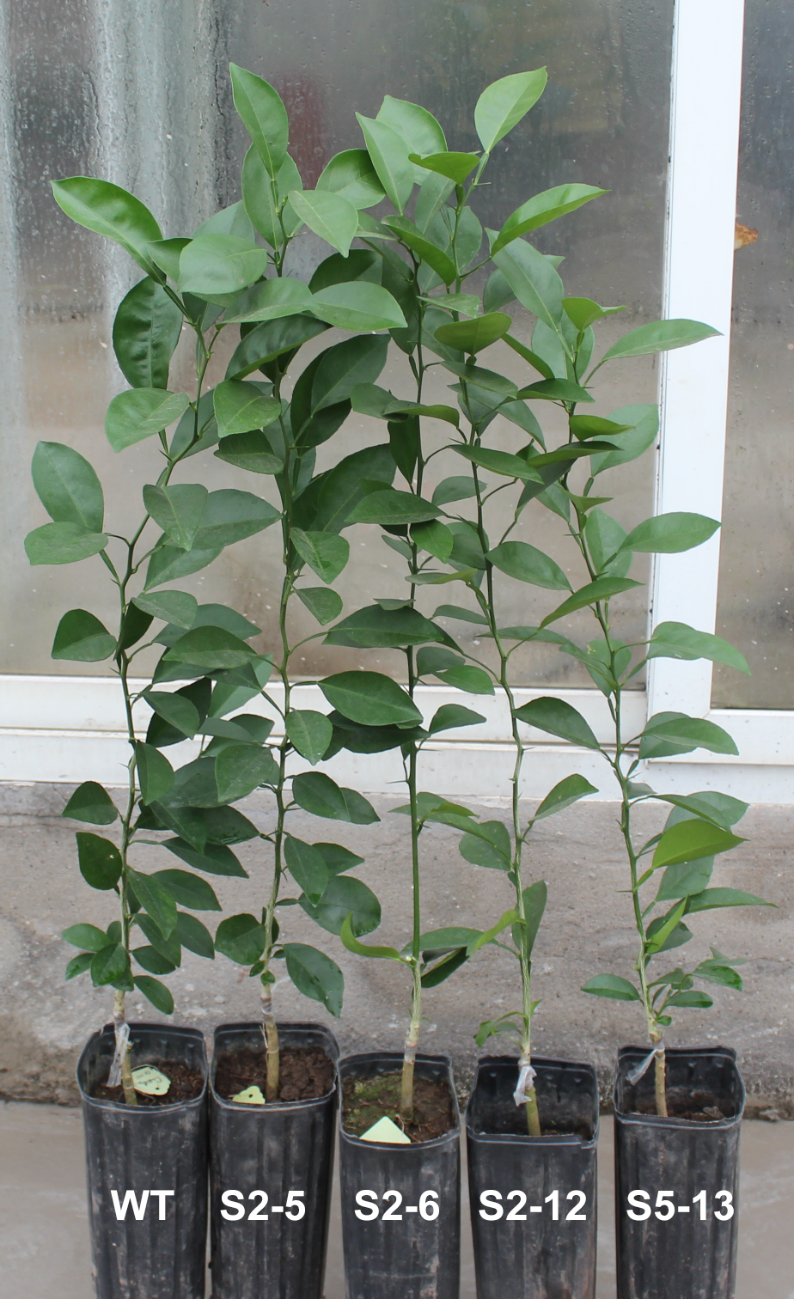


**Figure S9.** One-year-old modified plants growing in the greenhouse. No visible phenotypic difference was detected among the modified plants compared with the wild type (WT).


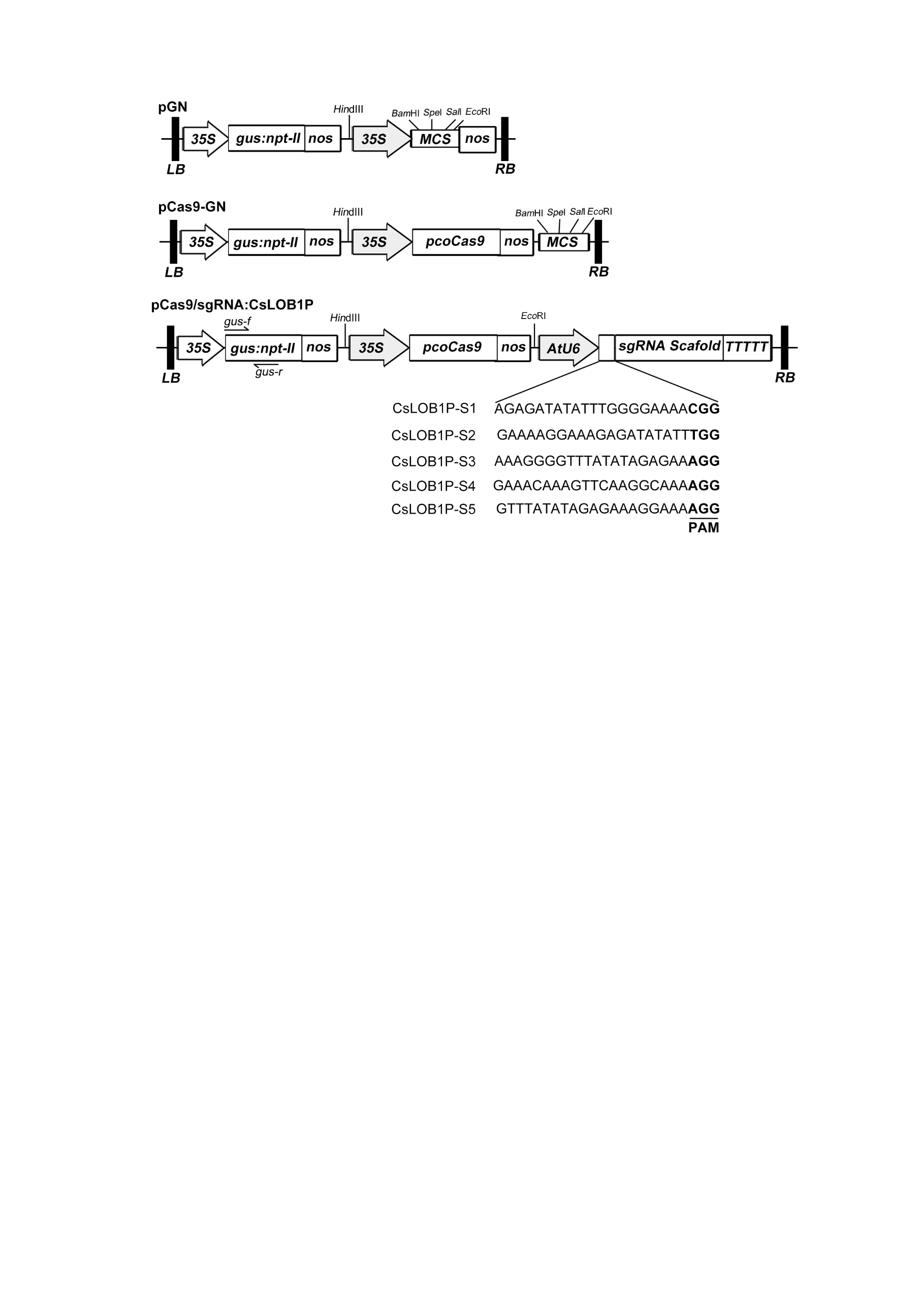


**Figure S10.** T-DNA structure of the PCas9-GN plasmid used in the study. The vector was constructed as follows: a *CaMV 35S* promoter with the *Hin*dIII/*Bgl*II restriction sites was amplified from the pGN plasmid (Zou et al., 2014) using 35S-f/35S-r primers (Table S6), lysed by the same enzymes and inserted into the *Hin*dIII/*Bam*HI-digested HBT-*pcoCas9* vector (Li et al., 2013). Next, the *pcoCas9* nuclease expression cassette driven by the *35S* promoter was cut from the vector using *Hind*III/*EcoR*I lysis (the *EcoR*I-end was blunted) and inserted into the *Hind*III/*Sma*I-digested pGN plasmid to generate the pCas9-GN vector. In the pCas9-GN vector, the *gus*::*npt-II* fusion gene was used as the selectable marker and reporter for the genetic transformation of citrus mediated by *Agrobacterium tumefaciens*. *35S*, *Cauliflower mosaic virus 35S* promoter from tobacco; *gus::npt-II*, fusion of β-glucuronidase and neomycin phosphotransferase genes (for screening of citrus transformants); *nos*, nos terminator; *pcoCas9*, a plant codon-optimized *SpCas9* (Li et al., 2013); MCS, multiple cloning sites; LB, left border; RB, right border.

**References**

Li, J.F., Norville, J.E., Aach, J., McCormack, M., Zhang, D., Bush, J., Church, G.M., et al. (2013) Multiplex and homologous recombination-mediated genome editing in *Arabidopsis* and *Nicotiana benthamiana* using guide RNA and Cas9. *Nat Biotechnol* **31**, 688-691.

Zou, X., Peng, A., Liu, q., He, Y., Wang, J., Xu, L., Lei, T., et al. (2014) Secreted Expression of cecropin B Gene enhances resistance to *Xanthomonas axonopodis* pv. *citri* in transgenic Citrus sinensis 'Tarocco'. *Acta Horticulturae Sinica* **41**, 417-428.
